# Entropic Control of Ethylene and Acetylene Uptake in Silicon-Functionalized Ionic Liquids? An Experimental and Molecular Dynamics Study

**DOI:** 10.1021/acs.jpclett.6c01430

**Published:** 2026-09-11

**Authors:** Eduards Bakis, Kateryna Goloviznina, Inês C. M. Vaz, Nithavong Cam, Agilio Padua, Margarida Costa Gomes

**Affiliations:** † Department of Chemistry, Faculty of Medicine and Life Sciences, 61769 University of Latvia, Jelgavas 1, Riga LV-1004, Latvia; ‡ Laboratoire de Chimie, ENS de Lyon and CNRS, 46 allée d’Italie, 69364 Lyon, France; § ICSM, University of Montpellier, CEA, CNRS, ENSCM, 30207 Bagnols-sur-Ceze, France

## Abstract

Distinguishing and rationalizing the physical absorption of small hydrocarbon gases is inherently challenging due to their nonpolar character. Similar interaction energetics obscure the molecular origin of solubility trends. Herein we combine isochoric saturation measurements and polarizable (CL&Pol) molecular dynamics simulations to examine ethylene absorption in seven silicon-functionalized imidazolium ionic liquids and extend the analysis to acetylene. Ethylene uptake increases with cation flexibility and free volume, with siloxane-functionalized cations and [NTf_2_]^−^ salts giving the highest solubility. Thermodynamic analysis shows that differences in ethylene solubility are driven mainly by entropy, consistent with cavity formation in nonpolar regions as the dominant uptake mechanism. Simulations reproduce these trends and reveal that acetylene, unlike ethylene, also benefits from specific solute-anion interactions, especially with more basic anions. These results provide clear design rules for reversible C2-hydrocarbon sorbents: increase cation flexibility to enhance ethylene absorption, and tune anion basicity to amplify acetylene uptake selectively.

## Graphical abstract

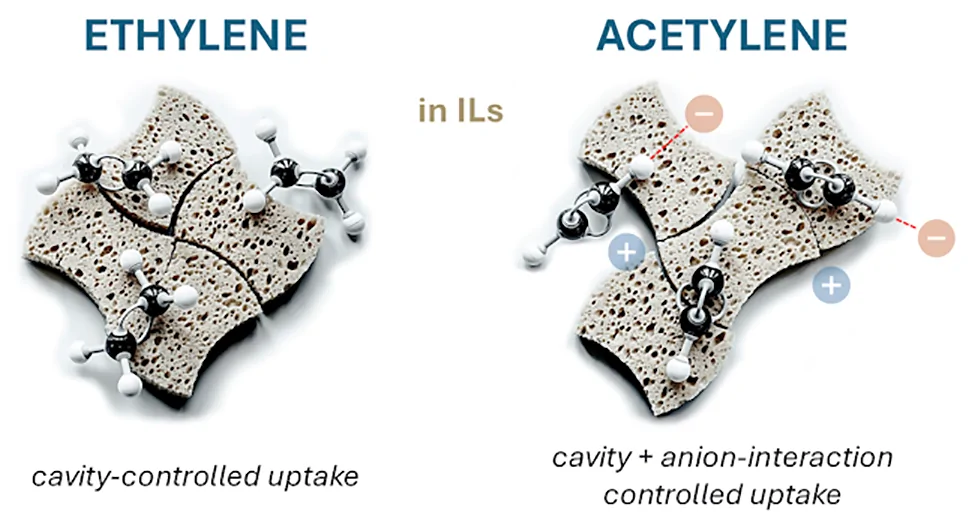

Small unsaturated hydrocarbons, such as ethylene and acetylene, are central to chemical manufacturing. Ethylene is a primary precursor to polyethylene, ethylene oxide, and numerous bulk chemicals,^1^ while acetylene remains a strategic feedstock for the Reppe process to butane-1,4-diol, which is used in the production of tetrahydrofuran.^2,3^ Interest in broader acetylene use has recently re-emerged.^4^ Because these gases are produced and used at scale, their selective, reversible absorption underpins key purification and handling steps.

Acetylene produced by an autothermal cracking process from natural gas is commonly isolated via fractional desorption from *N*,*N*-dimethylformamide or *N*-methylpyrrolidone^5^ owing to its high solubility in these solvents. By contrast, purification of ethylene from ethane is still performed predominantly by cryogenic distillation,^6^ one of the most energy-intensive separations in the chemical industry. Reducing the energy footprint of such gas purification steps is therefore a major industry target for innovation and climate change mitigation.^7–10^ Beyond petrochemicals and its value to the polymer industry, ethylene functions as a plant hormone regulating fruit ripening.^11^ Selective, reversible ethylene sorbents during storage and transport could also thus help manage ethylene,^12,13^ thereby reducing spoilage that accounts for significant amounts of food loss.^14^

Ionic liquids (ILs) are attractive designer materials for gas separations^15,16^ and chemical reactions, owing to their negligible volatility,^17^ structural tunability,^18^ and thermal stability.^19^ As reaction media, ILs can also enable homogeneous catalysis^20^ with gaseous reagents,^21^ offering levers unavailable in heterogeneous syntheses.^22^

Consequently, there is a strong incentive to understand the rules that govern gas solubility in ILs. Polar, reactive gases such as CO_2_ ^23^ and SO_2_ ^24^ are actively studied for reactive capture using ILs with chemical sites designed for this task. However, for weakly interacting gases such as C2 hydrocarbons, the extent to which gas solubility, and therefore sorption capacity and selectivity, can be systematically tuned by IL molecular design remains unresolved. Physical absorption of nonpolar gases in ILs is governed by diverse molecular forces:^25^ dispersion and short-range exchange-repulsion, permanent electrostatics, induction/many-body polarization, H-bonding, and interactions tied to quadrupole and π-electron density (e.g., dipole–quadrupole and cation-π contacts in the case of unsaturated gases). Because macroscopic gas-dissolution observables (Henry’s-law constants, heats of absorption, spectroscopic shifts) conflate these contributions, isolating their individual weights by experiment alone is challenging. Molecular simulations can contribute to the molecular understanding of the physisorption of gases in ILs.^26,27^

In this work, we focus on how silicon functionalization of IL cations can impact gas solubilization by understanding the balance between the aforementioned interactions. Silicon-containing motifs can increase side-chain flexibility, enhance polarizability, and create free volume,^28^ suggesting an enhanced route to favor weak reversible gas uptake without chemisorption. We therefore explored a family of seven Si functionalized ILs and a carbon-based counterpart by measuring low-pressure ethylene solubilities using the isochoric saturation method.^29^ The resulting Henry’s-law constants establish structure-solubility relationships between cation/anion variants and allow evaluation of dissolution thermodynamics, quantifying how enthalpic and entropic contributions evolve with molecular architecture. To characterize ethylene’s local solvation environment in ILs and decompose its solvation free energy into van der Waals, electrostatic, and polarization contributions, we performed molecular dynamics (MD) simulations with a state-of-the-art CL&Pol polarizable force field.^30–32^ As has been widely discussed in the literature,^33,34^ explicit representation of induction effects is essential for the correct prediction of the structure and dynamics of ILs. ILs containing alkylsilane and siloxane side groups have recently been included in CL&Pol,^28^ and in the present work we extend the model to new solute molecules. Finally, leveraging the validated ethylene description by MD, we predict acetylene solubility trends in the same ILs, measurements that can be hazardous and therefore are rarely performed.

## Ethylene Solubility

The molar fraction solubility of ethylene in the 8 ILs (Figure 1) was found to be on the order of 10^–2^, which is equivalent to 10 to 20 ethylene molecules per each 1000 ion pairs of the studied ILs at 0.1 MPa and 313 K. The calculated Henry law constants *K*_H_ are summarized in Table S4 of the Supporting Information and span from 5 MPa to around 13.5 MPa. By comparing the trends in ethylene solubility with those previously observed for argon^28^ - a noninteracting gas - it is possible to elucidate the role and significance of specific gas-IL interactions in governing the solubility behavior found herein.

**Figure 1:**
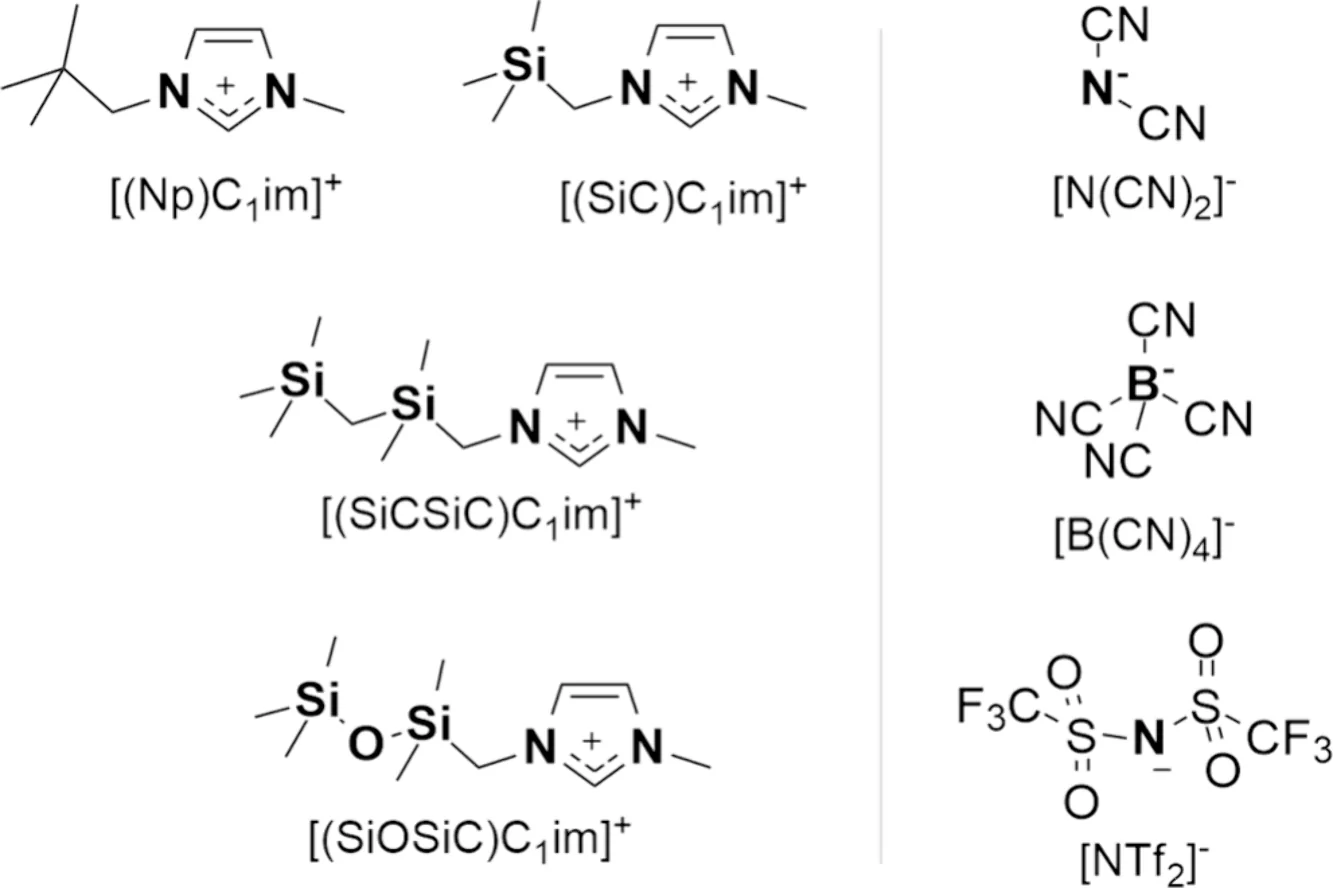
Structural formulas of the IL ions studied.

At 0.1 MPa and at all measured temperatures, the molar fraction solubility of ethylene (Figure 2) follows the trend for the ILs based on the [NTf_2_]^−^ anion: [(Np)­C_1_im]^+^ < [(SiC)­C_1_im]^+^ < [(SiCSiC)­C_1_im]^+^ < [(SiOSiC)­C_1_im]^+^. Generally, ethylene absorption correlates with the molar volume of the ILs, as expected for weakly interacting gases. This correlation is shown explicitly in the Supporting Information, where the ethylene mole-fraction solubility at 313 K and 0.1 MPa is plotted against the molar volume of the corresponding ILs (Figure S15). The positive correlation observed (R^2^ = 0.81) supports the interpretation that the ability of the liquid to accommodate a small nonpolar solute, through cavity formation/free-volume effects, is a major contribution to the observed solubility trend. Nevertheless, this correlation is approximate; and deviations from it indicate additional, more specific solute–solvent interactions introduced by cation/anion chemistry. It is worth noting that, contrary to what was seen in argon solubility,^28^ there is a measurable difference between the solubility of ethylene in [(SiC)­C_1_im]­[NTf_2_] and its carbon counterpart [(Np)­C_1_im] [NTf_2_], showing that substitution of carbon by silicon leads to an increase of the ethylene absorption capacity.

**Figure 2:**
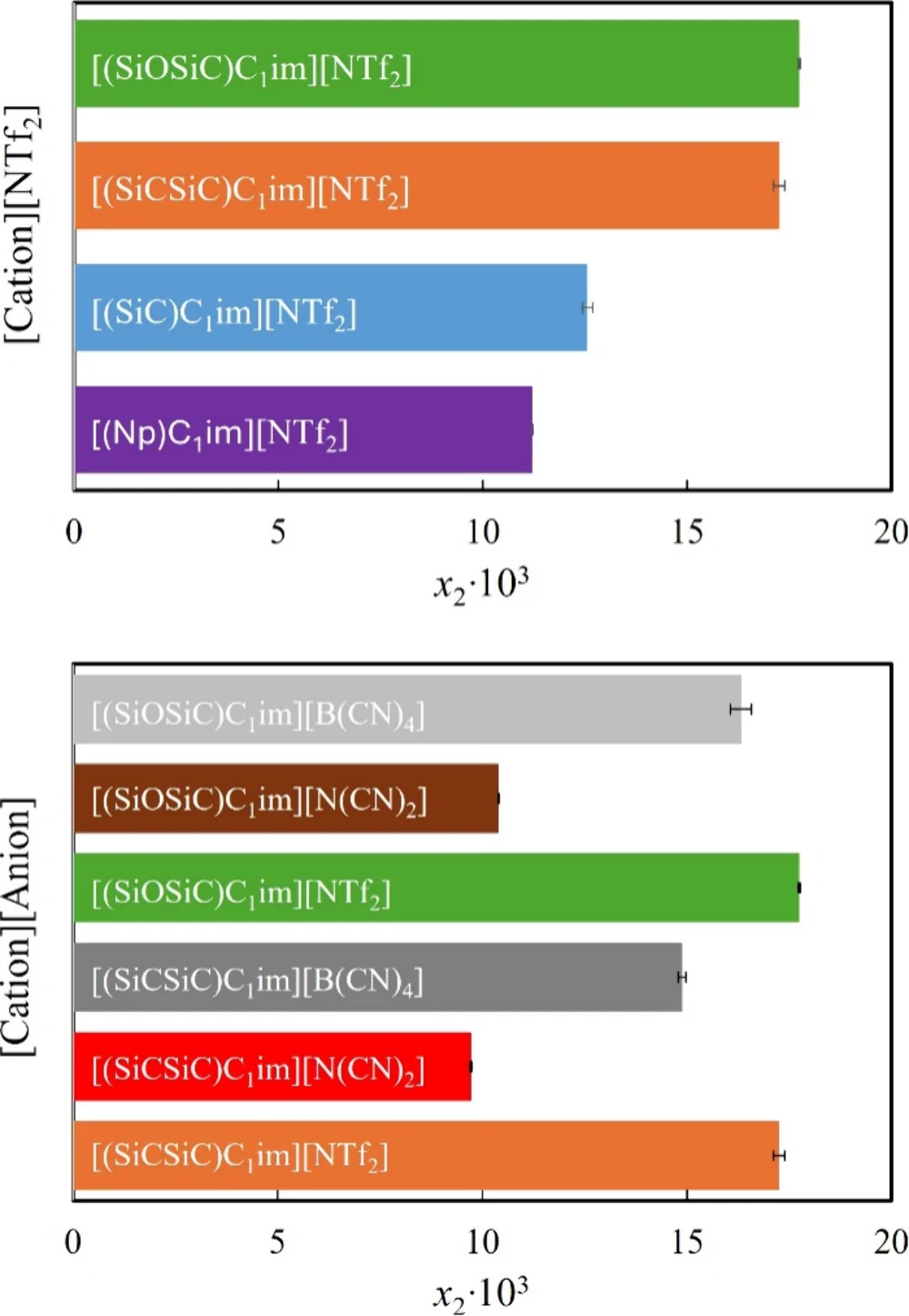
Solubility of ethylene in the studied ILs (top figure: [NTf_2_]^−^ ILs; bottom figure: ILs composed by different cations and anions) expressed as a molar fraction at 0.1 MPa pressure and 313 K. Error bars were estimated from the absolute relative deviation of the experimental data to second degree polynomial fits of solubility versus temperature.

Regarding the effect of the anion (Figure 2) within the ILs composed of the cations [(SiCSiC)­C_1_im]^+^ and [(SiOSiC)­C_1_im]^+^, at 0.1 MPa, the molar fraction solubility of ethylene follows the trend [N­(CN)_2_]^−^ < [B­(CN)_4_]^−^ < [NTf_2_]^−^. This trend corresponds exactly to what was observed for the argon solubility in these ILs.

It should also be noted that the solubility of ethylene in ILs based on the cation [(SiOSiC)­C_1_im]^+^ is always superior to that in those based on the cation [(SiCSiC)­C_1_im]^+^, independently of the anion that composes the IL. This behavior may be associated with the increased flexibility of the voluminous siloxane side chain.^35^

These data suggest that ethylene solubility is largely controlled by the entropic cost of forming a cavity in the IL, which decreases with increasing side-chain flexibility and free volume. Deviations from the argon-derived baseline, particularly upon Si substitution, point to additional weak specific interactions that we quantify below through solvation thermodynamics and MD simulations.

## Thermodynamics of Ethylene Solvation

The solubility of ethylene in the studied ILs can be rationalized by deriving the thermodynamic properties of solvation (see Supporting Information). As shown in Figure 3, the molar enthalpy of solvation at infinite dilution is consistently exothermic across all systems, with an average value of approximately −9.8 kJ·mol^–1^ and only minor variations between ionic liquids. Interestingly, this magnitude is comparable to that observable in molecular solvents such as benzene (−9.000 kJ·mol^–1^)^36^ and acetone (−9.071 kJ·mol^–1^)^36^ and is also within the range of the values reported for [C_4_C_1_im]­[NTf_2_] (the IL initially selected as a reference,^37^ values reported in the range from −11.9 kJ·mol^–1^ ^38^ to −9.0 kJ·mol^–1^ ^39^). Moreover, a study on the solvation of alcohols in [C_4_C_1_im]­[NTf_2_] estimated that each methylene group in alcohols contributes ≈ −4.4 kJ·mol^–1^ to the solvation enthalpy.^40^ The enthalpy of solvation of ethylene is similar in all of these different solvents (≈ −9 kJ·mol^–1^) and close to the sum of two −CH_2_– contributions (≈ −8.8 kJ·mol^–1^). All this suggests that, in this work, (i) ethylene solvation is largely governed by broad, nonspecific dispersive interactions; (ii) the cavitation effect should be similar across different solvents - and nearly negligible in terms of enthalpy cost - or at least offset to a comparable degree by solute–solvent interactions, regardless of the nature of the solvent.

**Figure 3:**
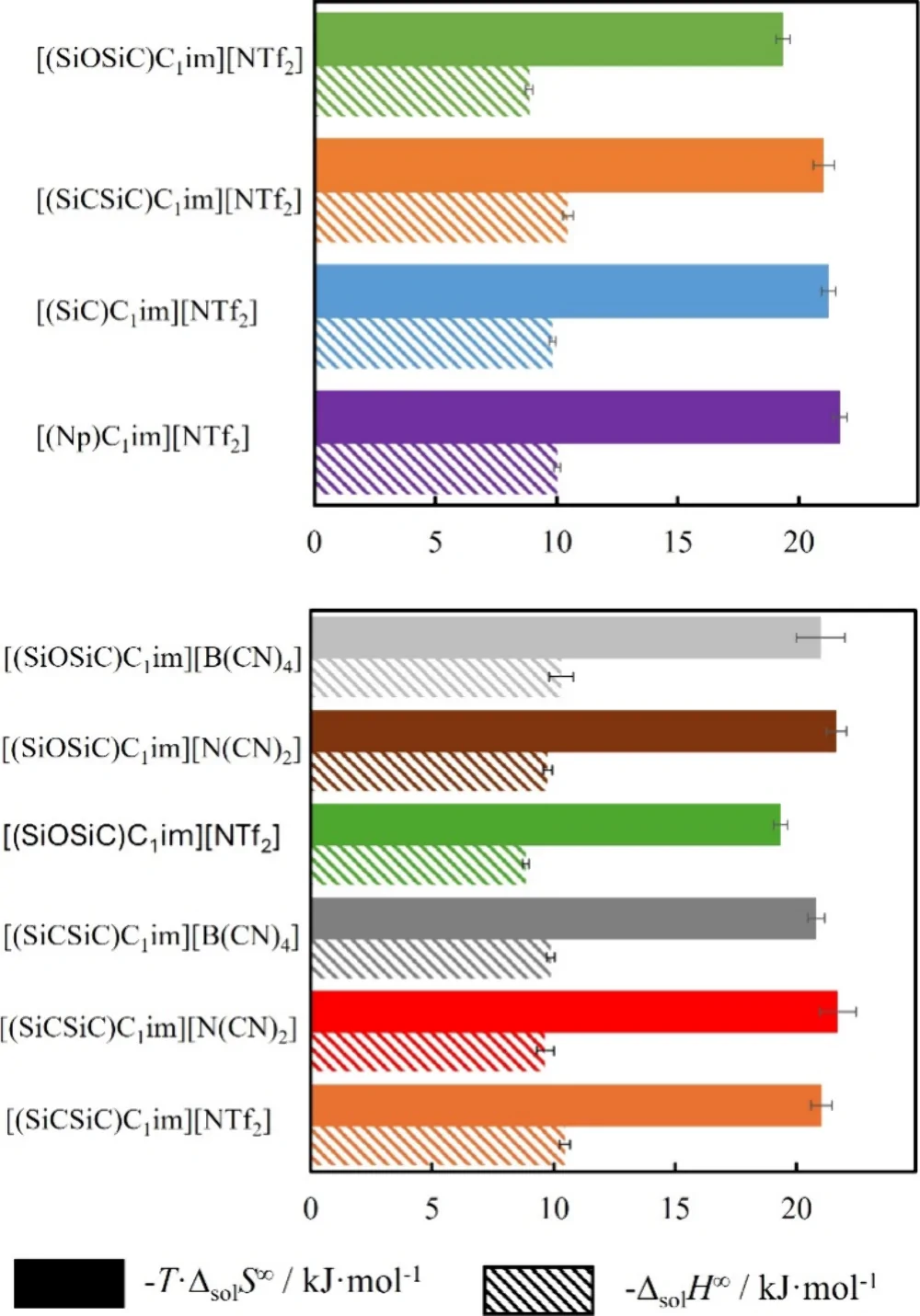
Standard molar enthalpy and entropy of solvation of ethylene in the studied ILs at infinite dilution (top figure: [cation]­[NTf_2_]; bottom figure: constituted by the cations [(SiCSiC)­C_1_im] and [(SiOSiC)­C_1_im] and the anions [NTf_2_], [N­(CN)_2_] and [B­(CN)_4_]) at infinite dilution at 313 K.

Although the overall free energy of ethylene solvation is dominated by the favorable enthalpic contribution, the entropy of solvation has an unfavorable contribution that shows a larger variation across the IL series. Consequently, despite the uncertainty associated with the thermodynamic analysis, significant differences in ethylene solubility correlate more consistently with changes in the entropy term than with changes in the enthalpy term. This relationship is illustrated in the Supporting Information by plotting the ethylene mole-fraction solubility at 313 K and 0.1 MPa against the entropic contribution to solvation (Figure S15). The resulting correlation is moderate (R^2^ = 0.63), supporting the role of solvation entropy as a useful descriptor while also indicating that entropy alone cannot account for all differences across the IL series.

The entropy of the ethylene solvation in benzene (−66.73 J·mol^–1^·K^–1^)^36^ and in acetone (−67.99 J·mol^–1^·K^–1^)^36^ is similar to the values obtained in the ILs studied. For [C_4_C_1_im]­[NTf_2_] IL, very different values have been reported for the entropy of ethylene solvation: −74.9 J·mol^–1^·K^–1^ ^38^ and −29.9 J·mol^–1^·K^–1^.^39^ Additionally, the molar entropy of solvation for argon^28^ at infinite dilution in the studied ILs is significantly more negative, ranging from −90 J·mol^–1^·K^–1^ to −115 J·mol^–1^·K^–1^, depending on the IL. The difference between ethylene and argon suggests that solvation is not determined solely by solute volume, but also by solute shape and compatibility with the solvent environment, results being compatible with a stronger entropic penalty for spherical nonpolar solutes as argon, in relation to more linear or planar molecules as ethylene.

This can be rationalized within the established concept of ILs as nanoscale heterogeneous liquids, composed of coexisting polar and nonpolar domains. Small nonpolar solutes such as ethylene may promote cavity formation within the nonpolar regions of the studied ILs under a solvent confinement less stringent than spherical argon, thereby lowering the entropic cost associated with solvent reorganization.

A conceptual framework for understanding the solvation of small nonpolar molecules can be drawn from cavity formation processes in cohesive liquids like water. In this context, the insertion of a small nonpolar solute requires local rearrangement of the surrounding medium to accommodate its presence, while maintaining overall intermolecular interactions within the liquid.^41^ A direct mechanistic comparison is of course not valid here, as ILs differ profoundly from molecular solvents (such as water) due to their amphiphilic character. Instead, this structural organization present in ILs provides distinct environments for solute accommodation, enabling preferential interactions of nonpolar molecules, such as ethylene, within nonpolar regions of the IL.

Within this framework, the nearly invariant enthalpy of solvation observed across the IL series suggests that ethylene incorporation does not significantly perturb the overall balance of the interaction energies. Instead, variations in solubility are more consistently reflected in the entropy of solvation, indicating that differences in solvation behavior are primarily associated with configurational freedom within the nonpolar domains and/or with variations in local reorganization.

In other words, as the entropy of solvation reflects the difference between the gaseous state and the solvated state, a less negative entropy may indicate that ethylene retains a higher degree of translational and/or rotational mobility within the nonpolar domains of the IL. Consistent with this interpretation, we suggest also that for the solvation of small, nonpolar solutes such as ethylene in the investigated ILs, the solvation entropy may also capture the degree of solvent reorganization needed to create cavities without significantly disturbing existing intermolecular interactions and/or the mobility of the solute within the nonpolar regions of the IL. The more negative entropies obtained for argon solvation - especially in ILs with a lower free volume and more basic anions, and thus a higher cohesive energy density - further corroborate this interpretation. On the basis of the current thermodynamic data, it is not possible to disentangle the individual contributions of these effects (variations in local reorganization and/or configurational freedom within the nonpolar domains).

Finally, the less negative entropy of ethylene solvation in the IL containing the [(SiOSiC)­C_1_im]^+^ cation and the [NTf_2_]^−^ anion (relative to the IL with [(SiCSiC)­C_1_im]^+^) provides additional support for the proposed interpretation. Despite the inherent uncertainties associated with the derivation of thermodynamic properties, which limit the interpretation of the solvation entropy for other anions, higher ethylene solubilities were found in all ILs containing the [(SiOSiC)­C_1_im]^+^ cation, regardless of the anion. We attributed this higher solubility (and less negative entropy of solvation) to the greater flexibility of the siloxane side chain. For the remaining anions, the associated uncertainties do not allow the same level of thermodynamic interpretation, although the solubility trends remain consistent with this hypothesis. The higher solubility of ethylene in [(SiC)­C_1_im]­[NTf_2_] than in its carbon counterpart [(Np)­C_1_im]­[NTf_2_] also seems to be explained by a less negative entropy of solvation, which also contributes to the previous interpretation.

Overall, ethylene solvation exhibits a nearly invariant exothermic enthalpy across the IL series, consistent with predominantly nonspecific dispersive interactions. It should be emphasized, however, that the present thermodynamic analysis does not allow for a quantitative decomposition of the absolute enthalpic and entropic contributions to solvation. Consequently, the experimental approach cannot provide unequivocal evidence for enthalpic or entropic control, and the derived thermodynamic parameters should be interpreted in a comparative rather than an absolute sense. Within these limitations, the observed differences in ethylene solubility correlate more clearly with variations in the entropy of solvation than with variations in the enthalpy of solvation, as also illustrated in Figure S15. However, the comparison with the IL molar volume shows an even stronger correlation, supporting the view that ethylene uptake is largely governed by cavity formation/free-volume effects. The entropy of solvation should therefore be interpreted as an additional descriptor, reflecting differences in solvent reorganization and/or in the configurational freedom retained by ethylene within the nonpolar domains of the ILs. While these observations do not constitute unequivocal evidence of entropic control, they are consistent with higher ethylene solubility in more flexible and higher free-volume systems.

## Solubility of Ethylene and Acetylene from MD Simulations

While the solubility of ethylene in the ILs discussed above can be determined experimentally, the obtaining of reliable acetylene solubility data remains challenging because of safety constraints. In this context, classical MD simulations offer a valuable alternative for probing the systems of interest: mole-fraction solubilities can be derived from the residual chemical potential, as described in the Supporting Information, and analyses of the local structure help rationalize the observed solubility patterns. To test the reliability of this strategy, we compared calculated ethylene solubilities in several ILs with silane and siloxane-based cationic side chains and different anions. As can be seen from our results (Figure 4a), classical MD simulations systematically underestimate ethylene solubility by about 20–35%, which rise from difficulties in converging free energy calculations as well as extrapolation of the experimental results to the MD simulation temperature. However, these discrepancies are considerably smaller than those previously reported for argon-based systems,^28^ where the experimental and calculated values differed by roughly a factor of 3. This improvement stems from the more structurally and chemically complex nature of ethylene, which engages in a wider variety of solute–solvent interactions that extend beyond simple van der Waals forces. The experimental solubility trend along the anionic series with the values decreasing in the row [NTf_2_]^−^ > [B­(CN)_4_]^−^ > [N­(CN)_2_]^−^ (Figures 4a and 2) is well reproduced. The predicted values for [(SiCSiC)­C_1_im]^+^ and [(SiOSiC)­C_1_im]^+^ cation are rather close, but this trend is more in agreement with the experimental results measured at 313 K than extrapolated to 353 K.

**Figure 4:**
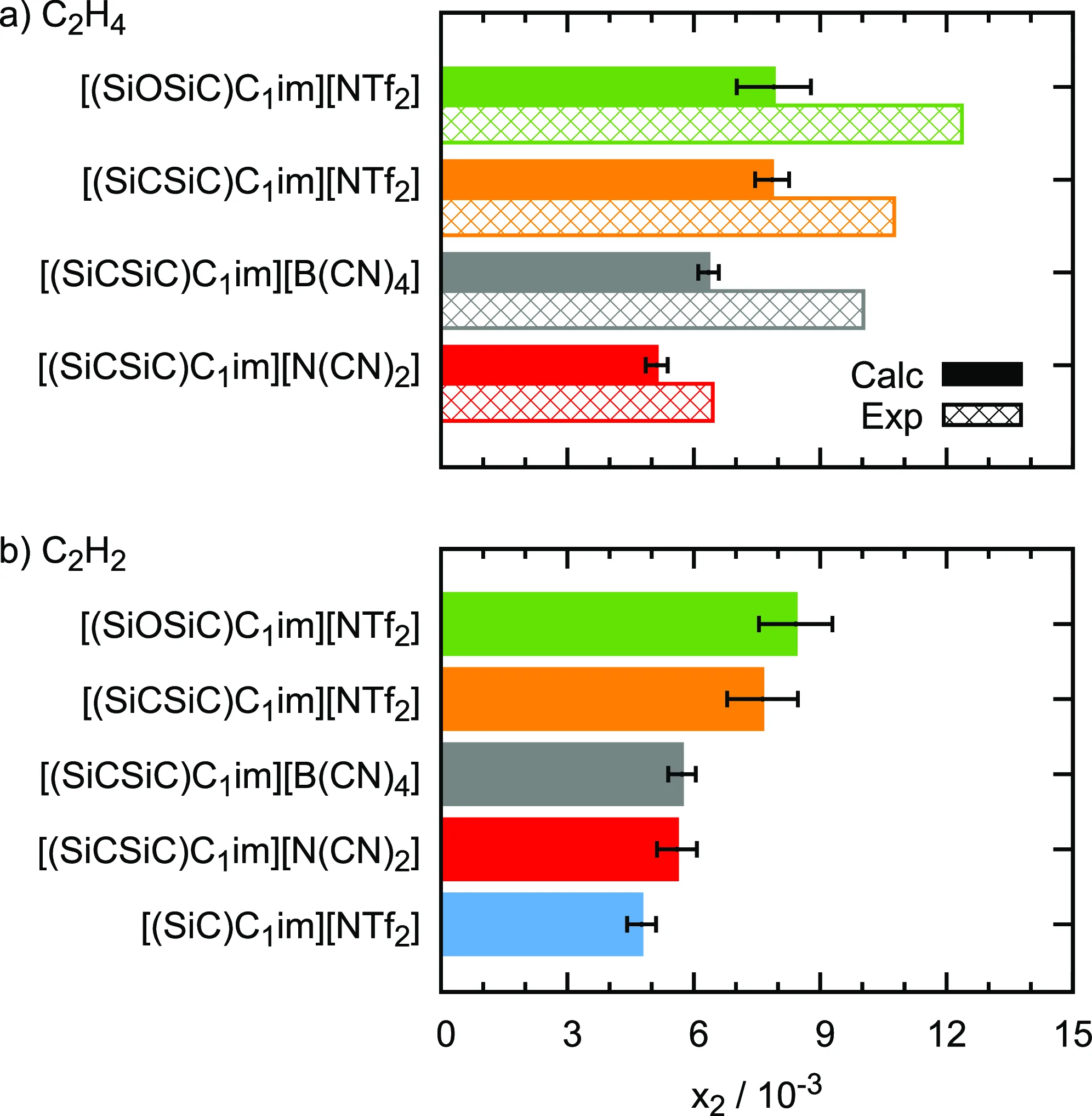
Calculated molar fraction solubilities (*x*_2_) of ethylene (a) and acetylene (b) in selected ionic liquids at a pressure of 0.1 MPa and 353 K, compared to the extrapolated experimental values. The corresponding error bars were computed from independent free energy calculation runs, with the details on calculation protocol provided in Supporting Information.

The calculated acetylene solubilities (Figure 4b) in the ILs series containing the [NTf_2_]^−^ anion shows that the influence of the cation mirrors that observed for argon and ethylene: solubility increases with the growth of the free volume, following the order [(SiC)­C_1_im]^+^ < [(SiCSiC)­C_1_im]^+^ ≲ [(SiOSiC)­C_1_im]^+^. Within the set of ILs based on the [(SiCSiC)­C_1_im]^+^ cation paired with various anions, the [NTf_2_]^−^-containing IL again exhibits the highest acetylene solubility, consistent with the behavior found for ethylene and argon. By contrast, ILs with the anions [B­(CN)_4_]^−^ and [N­(CN)_2_]^−^ display almost identical solubilities, and the slight enhancement in the [N­(CN)_2_]^−^ system may stem from specific anion-solute interactions, which will be examined in more detail further.

## Insight into the Solvation Mechanism and Local Structure

To interpret the observed solubility trends, we decomposed the MD-computed residual chemical potential into separate contributions: van der Waals, electrostatic, and polarization. As can be seen from Figure 5, the van der Waals term for gas solvation is always positive, associated with the energy cost to create a cavity, while electrostatic and polarization terms are negative, favored by solute interactions with ionic solvent species. For ethylene (Figure 5a), the van der Waals term plays the major role, decreasing in the row [(SiCSiC)­C_1_im]­[B­(CN)_4_] > [(SiCSiC)­C_1_im]­[N­(CN)_2_] > [(SiCSiC)­C_1_im]­[NTf_2_].

**Figure 5:**
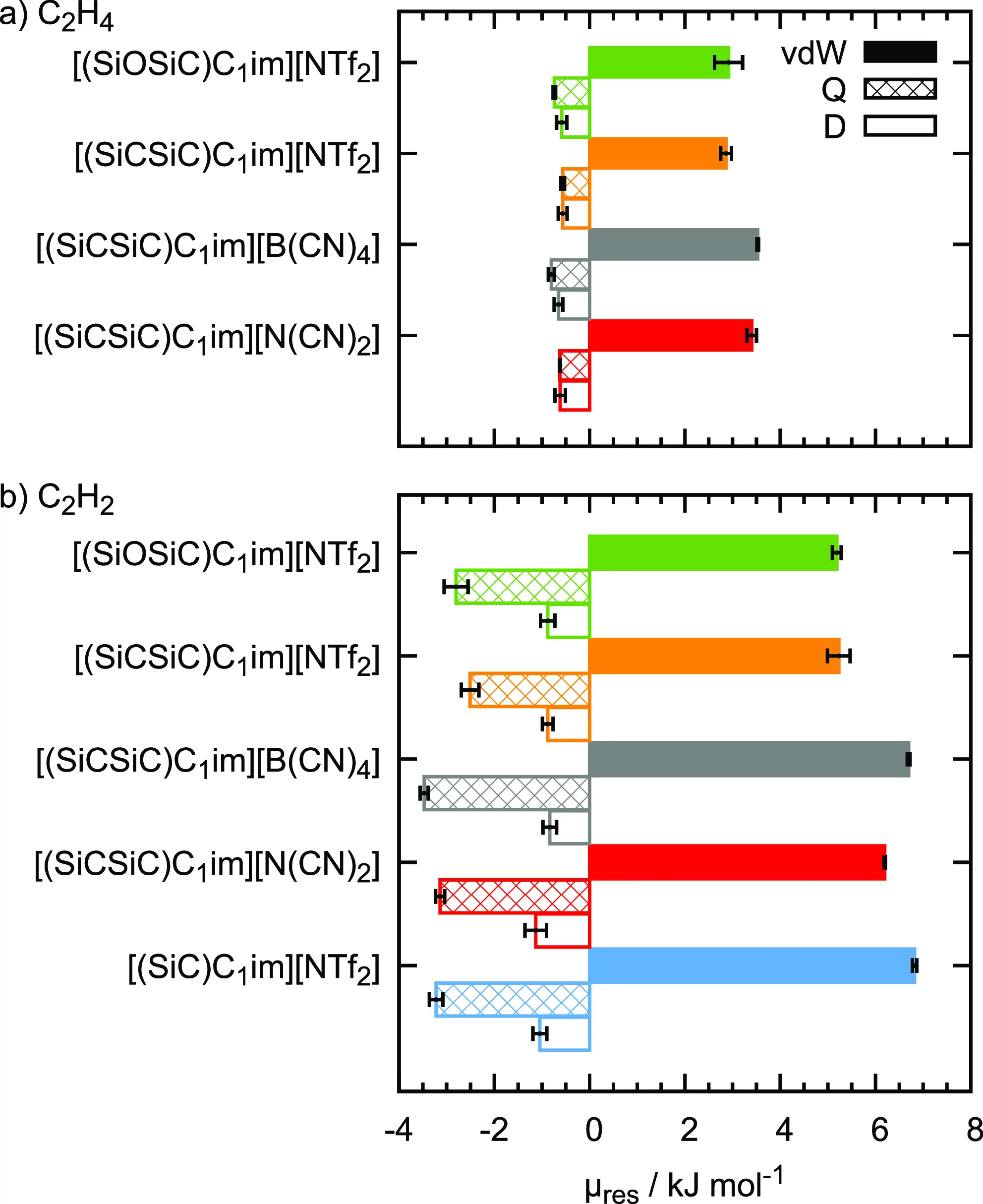
Decomposition of the MD computed residual chemical potential (μ_res_) of ethylene (a) and acetylene (b) in selected ionic liquids into van der Waals (vdW, solid filling), electrostatic (charge, *Q*, cross-diagonal hatch filling), and polarization (dipole, *D*, empty filling) terms. The corresponding error bars were computed from independent free energy calculation runs, with the details on calculation protocol provided in Supporting Information.

The radial distribution functions (Figure 6a and S5) demonstrate the cavity where the hydrocarbon is solvated is predominantly hydrophobic, shaped mainly by Si-containing side chains, with a smaller contribution from CH_3_–N cationic groups and, when present, from the CF_3_ groups of the [NTf_2_]^−^ anion. This agrees with our earlier argon study.^28^ The electrostatic and polarization contributions are considerably smaller, arising from minor π-stacking between the solute C=C bond and the imidazolium headgroup (Figure 7b, S6–9), which was already reported using quantum calculations of [Bmim]^+^...C_2_H_4_ dimers,^42^ and no additional specific solute–solvent interactions are detected.

**Figure 6:**
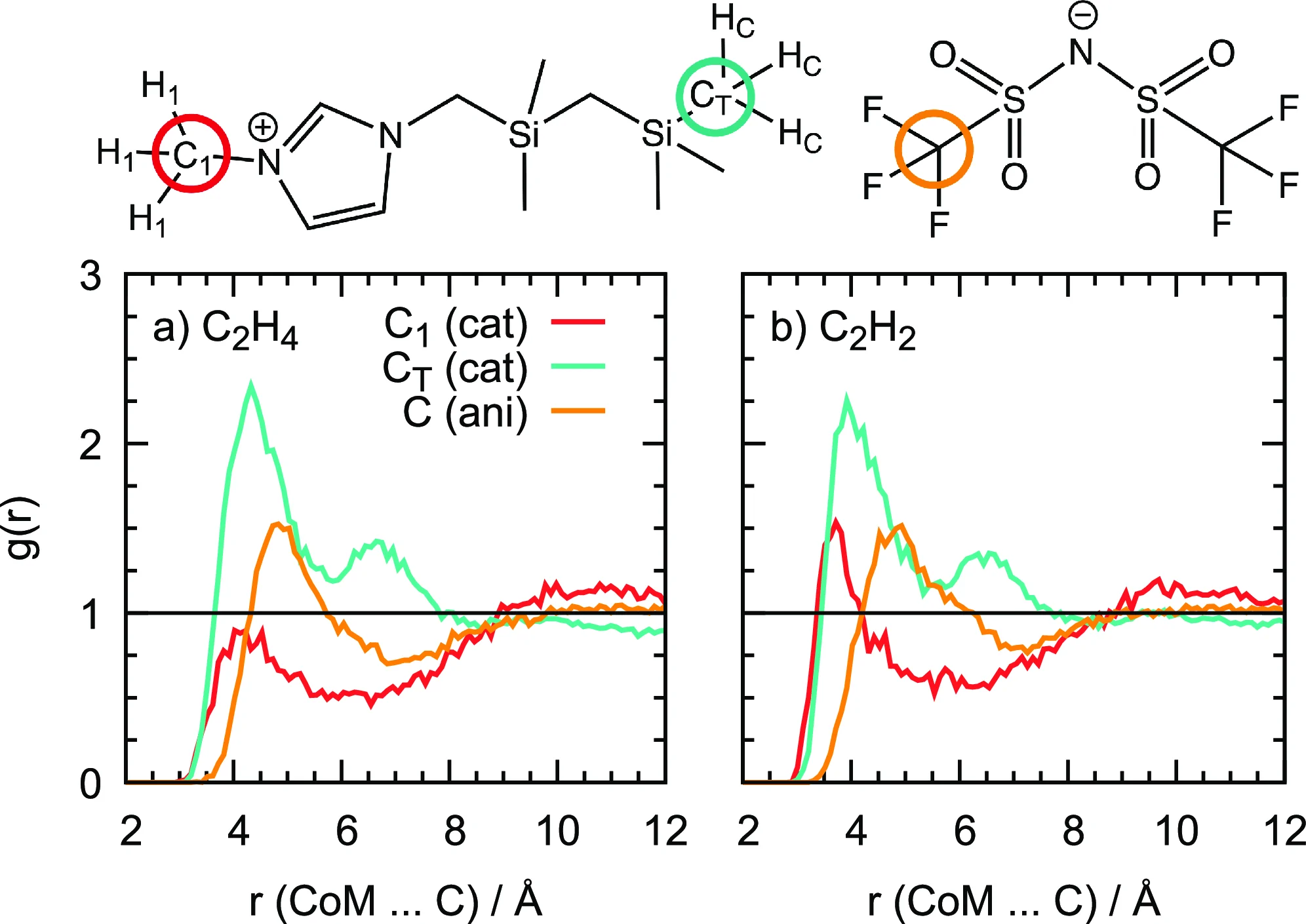
Radial distribution functions, g­(r), of selected carbon atoms of [(SiCSiC)­C_1_im]­[NTf_2_] ionic liquid around the center of mass (CoM) of ethylene (a) and acetylene (b).

**Figure 7:**
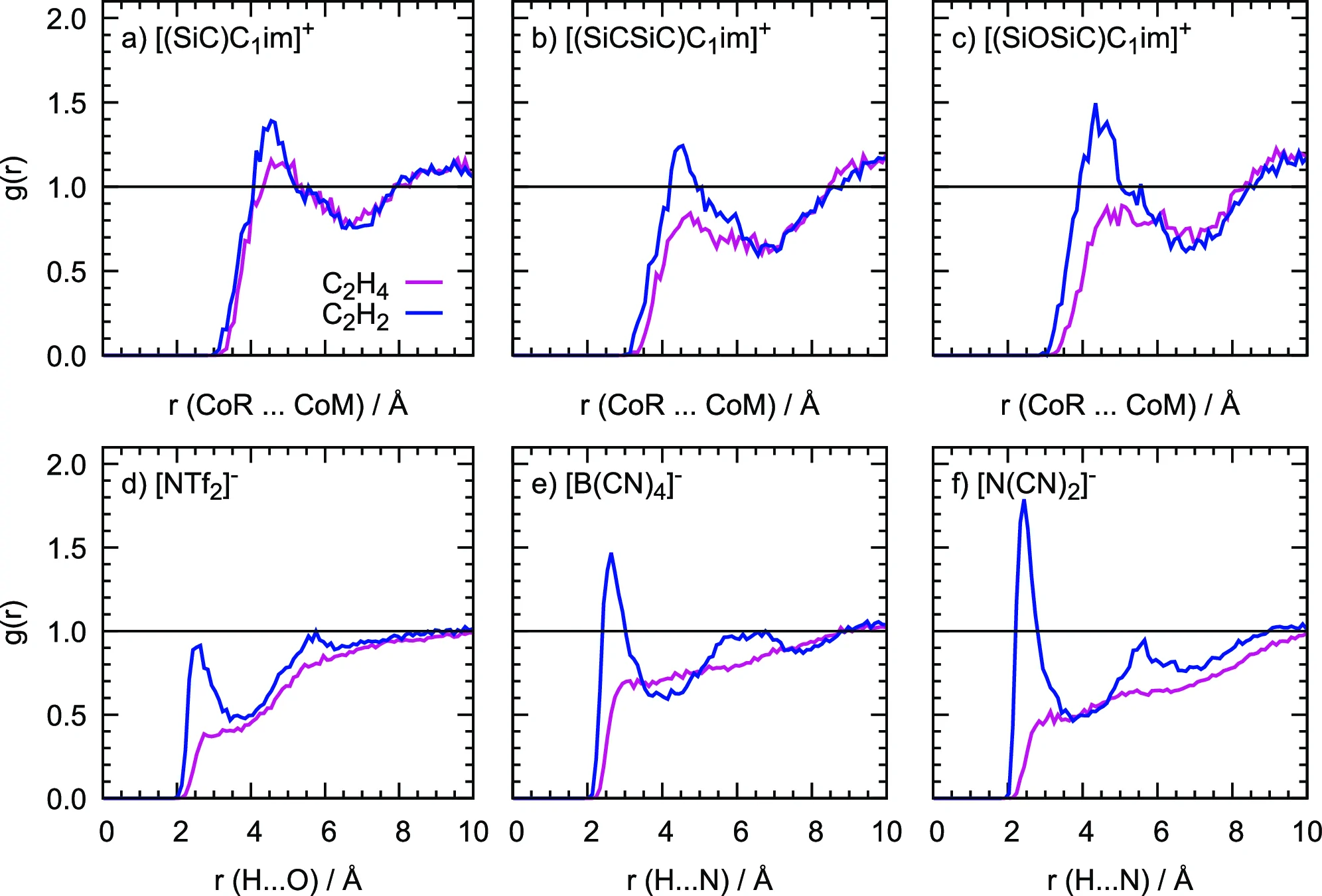
Radial distribution functions, g­(r), of ethylene (rose line) and acetylene (blue line) around selected atoms of ILs. Top: center of mass (CoM) of gas molecules around the center of ring (CoR) of the imidazolium headgroup in [(SiC)­C_1_im]­[NTf_2_] (a), [(SiCSiC)­C_1_im]­[NTf_2_] (b) and [(SiOSiC)­C_1_im]­[NTf_2_] (c). Bottom: hydrogen atoms of solutes around oxygen atoms of [NTf_2_]^−^, terminal nitrogen atoms of the [B­(CN)_4_]^−^ and [N­(CN)_2_]^−^ anions in [(SiCSiC)­C_1_im]­[NTf_2_] (d), [(SiCSiC)­C_1_im]­[B­(CN)_4_] (e), and [(SiCSiC)­C_1_im]­[N­(CN)_2_] (f) ionic liquids.

In the case of acetylene (Figure 5b), the van der Waals contribution increases by a factor of 1.7–2.6 compared to ethylene, explained by steric effects due to the more linear and slightly longer shape of acetylene. The electrostatic and polarization terms, in absolute values, are also notably greater due to larger partial charges on the acetylene H atoms and consequently their higher acidity. Compared to ethylene, a more pronounced π-stacking between the unsaturated C≡C bond of the solute and the imidazolium headgroup can be observed for acetylene (Figures 7a-c, S5, and S7–S9). Nevertheless, this interaction still remains minor due to acetylene preferential solvation by Si-containing side chains (Figure 6b). The cationic effect manifests itself as a reduction in the van der Waals contribution when moving from the smaller to the larger cation, [(SiC)­C_1_im]^+^ > [(SiCSiC)­C_1_im]^+^ ≳ [(SiOSiC)­C_1_im]^+^, which aligns with the observed free-volume trend. The electrostatic component follows the order [(SiC)­C_1_im]^+^ > [(SiOSiC)­C_1_im]^+^ > [(SiCSiC)­C_1_im]^+^ (in terms of absolute magnitude), and the polarization contribution is also strongest for [(SiC)­C_1_im]^+^ compared with the other two cations. Nonetheless, this behavior does not completely mirror the π-stacking probabilities (Figure 7) or free volume trends, indicating that additional factors are at play.

Within the anion series, the van der Waals contribution is largely governed by anion flexibility: for the bulky, rigid [B­(CN)_4_]^−^ and the more compact [N­(CN)_2_]^−^, the formation of a cavity requires more free energy than for the similarly large but highly flexible [NTf_2_]^−^. This effect is counterbalanced by specific solute–solvent interactions - electrostatic and polarization contributions, often described as H-bonding - between the anions and the acidic acetylene hydrogens. The likelihood of such formation of H-bonds (Figure 7d-f) increases along the sequence [NTf_2_]^−^ < [B­(CN)_4_]^−^ < [N­(CN)_2_]^−^, reflecting the rising basicity of the anions.^43^ For [N­(CN)_2_]^−^, acetylene tends to abandon the π-complex with the imidazolium cation (Figure S6c) in order to gain a more favorable interaction with the anion. This accounts for the observation that acetylene solubility in [(SiCSiC)­C_1_im]­[N­(CN)_2_] is nearly as high as in [(SiCSiC)­C_1_im]­[B­(CN)_4_] (Figure 4b), unlike ethylene, which does not engage in H-bonding with the solvent (Figure 4a, 7e–f). Consequently, some anions are effectively pulled away from the cation by acetylene, leaving free CH_3_–N cationic groups that enhance the hydrophobic character of the cavity (Figure 6b) more than in the ethylene-based system.

Overall, the ethylene benchmark demonstrates that the polarizable MD simulations capture solubility trends, enabling predictive estimates for acetylene, where experiments are impractical. Mechanistically, ethylene uptake is dominated by cavity formation (van der Waals/free-volume control), whereas acetylene introduces an additional chemical lever, anion basicity, through specific C–H···anion interactions, which with π-stacking can enhance solubility in selected ILs.

In conclusion, we systematically investigated the solubility structure–property trends of ethylene and acetylene in a series of 8 imidazolium-based ILs with varying side chain flexibility and corresponding free volume, using an integrated experimental - modeling approach.

The experimental solubility of the ethylene results, together with previously reported data for argon in the same ILs, provide mechanistic insights into the solvation and, particularly, into the cavitation mechanisms present. Considering that cavitation depends on the interplay between the internal pressure of the solvent and its cohesive energy density, as in previous studies,^41,44^ our results indicate that, in the ILs studied herein, the “creation of a cavity” for the solvation of ethylene (or argon) is most consistent with occurring through a rearrangement of the solvent species with minimal disruption of intermolecular interactions. The comparatively less negative entropy observed for ethylene relative to argon likely reflects a combination of effects, including (i) a lower degree of solvent structural constraint owing to the shape and nature of the ethylene and (ii) a higher residual mobility within the nonpolar domains of the ILs. The present data does not allow us to unambiguously separate these contributions, and both likely contribute to the observed entropic differences. Considering the weakly interacting character of ethylene, the enthalpy of solvation is essentially invariant within uncertainty across the ILs studied, indicating a similar cavitation mechanism and the dominance of nonspecific dispersive (van der Waals) interactions with the nonpolar regions of the ILs. The combination of enthalpic similarity and entropic variation observed in the studied systems suggests that, while solvation is largely enthalpically dominated, differences in solubility among ILs can be tuned by modulating the entropic contributions. This highlights the important role of strategies that increase solvent flexibility, such as the introduction of alkyl, silane, or siloxane motifs.

MD simulations with the CL&Pol polarizable model captured the experimentally observed solvation mechanism of ethylene in the ILs. They also enabled assessment of solubility trends for hazardous acetylene, which was not accessible experimentally, with the molar fraction solubility values predicted to be of the same order of magnitude as those for ethylene. By isolating van der Waals, electrostatic and polarization terms, we revealed their larger contributions (by absolute values) to the solvation free energy of acetylene than of ethylene, consistent with its longer and linear shape, and greater partial charges. The latter resulted in the formation of H-bonds between acetylene and IL anions, as well as enhanced (comparing to ethylene) π-stacking with the imidazolium cation. Accordingly, acetylene solubilities experience an impact of anion basicity, which is not present in the case of ethylene.

Overall, this work identifies molecular-level principles governing reversible absorption of small unsaturated hydrocarbons in structurally diverse ILs and establishes molecular guidelines for next-generation sorbents: increasing the cation flexibility/free volume to increase ethylene uptake and exploiting the anion basicity to promote specific interactions that preferentially increase acetylene absorption.

## Experimental Methods

The synthesis and characterization of the ILs studied herein have been described by us before.^28^

### Gas Solubility Measurements

The ethylene used in the gas solubility measurements was supplied by *Air Liquide* at a purity level of 99.95%. Its absorption measurements were made via the volumetric isochoric saturation method, as described previously.^29^ However, a miniaturized, all-stainless-steel apparatus was used, details of which are provided in the Supporting Information, Section 4.1. The accurate volume of this cell and its components was determined prior to the solubility measurements from the expansion of argon of a glass bulb whose volume is accurately known.

During the experiments, ethylene gas was placed in contact with a precisely measured (gravimetrically) amount of degassed IL at a constant temperature. When the vapor pressure above the liquid solution became constant, it was assumed that thermodynamic equilibrium had been reached, with the corresponding pressure denoted as the pressure in equilibrium, *p*_eq_. The amount of solute absorbed, *n*_2_^liq^, and consequently the solubility of the gas, is determined from the difference between two PVT measurements, as described in the following equation:

$$n_{2}^{\mathrm{liq}}=\frac{p_{\mathrm{ini}}\cdot V_{\mathrm{ini}}}{Z_{2}\left(p_{\mathrm{ini}},T_{\mathrm{ini}}\right)\cdot R\cdot T_{\mathrm{ini}}}-\frac{p_{\mathrm{eq}}\cdot {(V}_{\mathrm{tot}}-V_{\mathrm{IL}})}{Z_{2}\left(p_{\mathrm{eq}},T_{\mathrm{eq}}\right)\cdot R\cdot T_{\mathrm{eq}}}$$

As shown in the previous equation, the amount of solute absorbed is calculated from (i) the total amount of gas molecules determined from the initial pressure of the gas, *p*_ini_, at the initial temperature *T*_ini_, when it is introduced into the vapor phase chamber (VC) of accurately known volume *V*_ini_; and (ii) the amount of gas molecules that remain unabsorbed (that is, those that stay in the gas phase, after the gas expands into the full metallic cell and thermodynamic equilibrium with the IL is established). Here, the pressure and temperature of the metallic cell are denoted by *p*_eq_ and *T*_eq_; once equilibrium between ethylene and the IL is achieved, *V*_tot_ and *V*_IL_ correspond to the total volume of the metal cell and the volume occupied by the IL, respectively. *Z*_2_ is the compressibility factor of the gas in the metal-cell.

In the methodology described here, the vapor pressure of the studied ILs was negligible, and the volume of the IL was considered to not change upon gas absorption. Further justification regarding the assumptions of negligible IL vapor pressure and negligible liquid volume change upon ethylene absorption is provided in the Supporting Information, Section 4.2. The determined solubility results are expressed as the molar fraction of the gas in the IL or as Henry’s law constants, *K*_H_, as follows

$$x=\frac{n_{2}^{\mathrm{liq}}}{n_{2}^{\mathrm{liq}}+n_{1}^{\mathrm{liq}}}$$

$$K_{H}=\underset{x_{2}\to 0}{\lim}\frac{f_{2}\left(p_{eq},T_{\mathrm{eq}},x_{2}\right)}{x_{2}}\approx \frac{\phi_{2}\left(p_{eq},T_{\mathrm{eq}}\right)\cdot p_{\mathrm{eq}}}{x_{2}}$$

where *f*_2_ is the gas fugacity and ϕ_2_ is its fugacity coefficient, calculated using data from the second virial coefficient. The solubility data, the evaluation of its precision, and the changes with temperature, as well as the description of the derivation of the corresponding thermodynamic properties, are described in the Supporting Information.

### Molecular Dynamics Simulations

Classical molecular dynamics simulations were performed in the LAMMPS software.^45^ For ILs, a previously developed polarizable CL&Pol force field was used.^28^ For ethylene and acetylene, nonpolarizable OPLS-AA model^46^ was transformed into a polarizable one following the CL&Pol methodology.^30,31^ Polarization effects were represented explicitly using Drude induced dipoles.^47,48^ Cubic periodic simulation boxes, containing several molecules of unsaturated C2 hydrocarbons and 100–200 solvent ion pairs, were equilibrated for 2 ns at 353 K and 1 bar and followed by production runs of tens of ns. Local structure analysis was performed on 20 ns equilibrium trajectories. Free energy perturbation (FEP) method was used to compute the residual chemical potential (μ_res_) of ethylene and acetylene in ILs using a thermodynamic cycle (Figure S3) from separate trajectories. The solute–solvent interactions were consecutively turned on/off in a given IL and in vacuum, so that μ_res_ was computed as chemical potential difference between two states

$$\mu_{\mathrm{res}}=\underset{i}{\sum }\left({\Delta \mu }_{i}^{\mathrm{IL}}-{\Delta \mu }_{i}^{\mathrm{vac}}\right)$$

with *i* referring to van der Waals, electrostatic, and polarization interaction terms. The gas solubility *x* was then evaluated as

$$x_{2}=\frac{p^{*}}{\rho_{\mathrm{IL}}RT}\exp\left(-\frac{\mu_{\mathrm{res}}}{RT}\right)$$

where ρ_IL_ is the molar density of the IL solvent and p* is the partial pressure of the gas (0.1 MPa). A detailed description of the simulation protocol and the force field development procedure can be found in Supporting Information.

## Supplementary Material



## Notes

Experimental data on gas solubility measurements, including description of the metal absorption cell, equilibrium data, derivation of thermodynamic data, and estimation of uncertainties; details on molecular dynamics simulations: simulation protocol, force field development and validation, and free energy calculations (PDF)

## ∥

CICECO – Aveiro Institute of Materials, Department of Chemistry, University of Aveiro, 3810-193 Aveiro, Portugal
