# Supplementary material for: Entropic Control of Ethylene and Acetylene Uptake in Silicon-Functionalized Ionic Liquids? An Experimental and Molecular Dynamics Study
Source: J Phys Chem Lett. 2026 Sep 11;17(38):10783–91. doi: 10.1021/acs.jpclett.6c01430 (PMC13618048; doi:10.1021/acs.jpclett.6c01430)
Supplement: Supplementary file 1 [file jz6c01430_si_001.pdf]

Entropic Control of Ethylene and Acetylene Uptake in Silicon-Functionalized Ionic Liquids? An Experimental and Molecular Dynamics Study

Eduards Bakis,*[a] Kateryna Goloviznina,*[bc] Inês C. M. Vaz,[b§] Nithavong Cam,[b] Agilio Padua[b] and Margarida Costa Gomes[b]*

[a] *Department of Chemistry, Faculty of Medicine and Life Sciences, University of Latvia, Jelgavas 1, Riga, LV-1004, Latvia*

[b] *Laboratoire de Chimie, ENS de Lyon and CNRS, 46 allée d'Italie, 69364 Lyon, France*

[c] *ICSM, University of Montpellier, CEA, CNRS, ENSCM, 30207 Bagnols-sur-Ceze, France*

[§] *Present address: CICECO – Aveiro Institute of Materials, Department of Chemistry, University of Aveiro, 3810-193 Aveiro, Portugal*

Supporting Information

1. Ion structures and nomenclature for ionic liquids studied	**2**
2. Preparation of ionic liquids	**3**
3. MD simulation	**3**
3.1 Simulation protocol	**3**
3.2 Force field development	**4**
3.3 Free energy calculations	**7**
3.4 Simulation results	**9**
4. Gas solubility measurements	**12**
4.1 Description of the new metallic cell	**12**
4.2 Gas solubility results	**13**
4.3. Thermodynamic properties derived from gas solubility measurements	**20**
References	**25**

1. Ion structures and nomenclature for ionic liquids studied

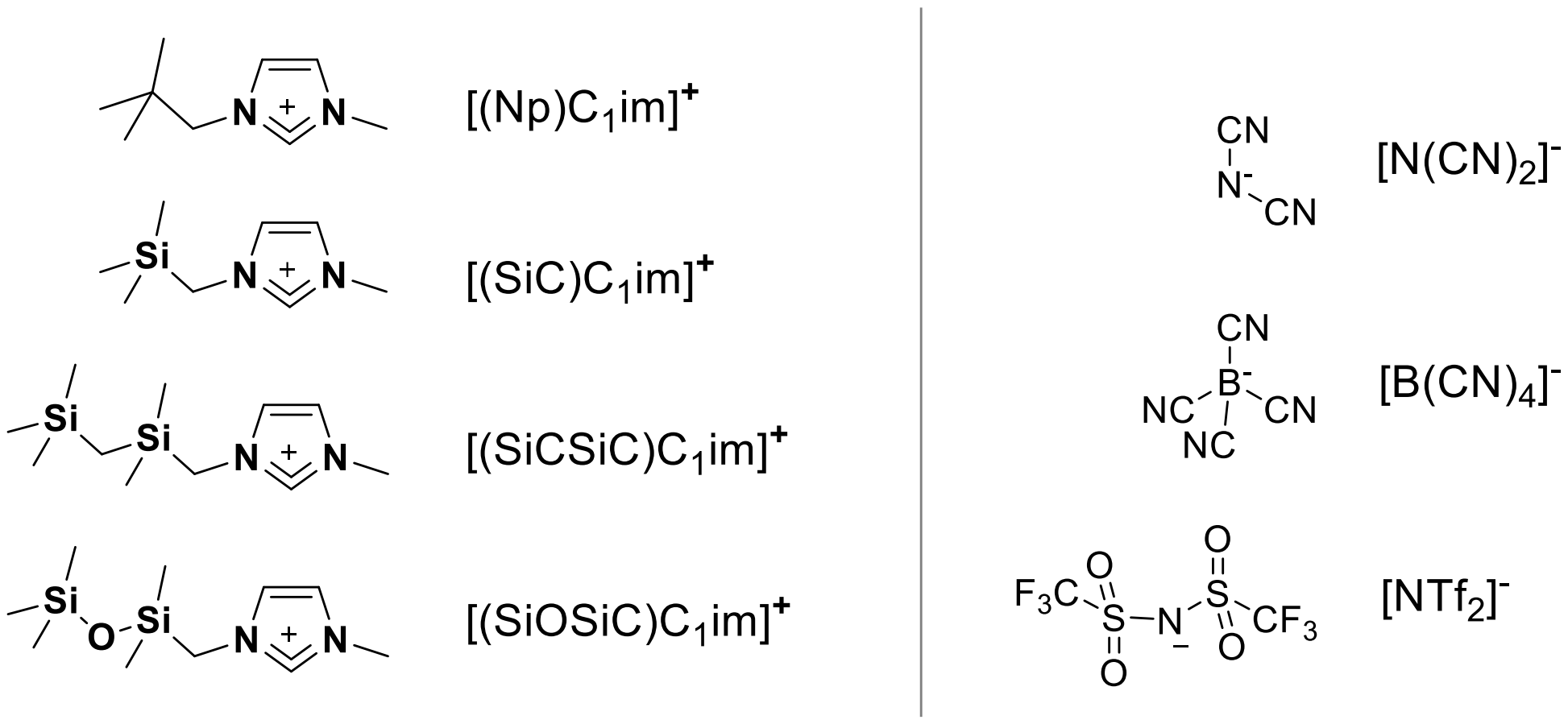

Scheme S1. Schematic structures and abbreviations of the ionic liquid anions and cations studied in this work

Table S1. List of the Ionic liquids studied in this wok, the CAS registry numbers and its molecular weight (g mol^{-1}).

No	Ionic Liquid	CAS No	MW
**1**	[(Np)C_1im][NTf_2]	871127-69-4	433.3908
**2**	[(SiC)C_1im][NTf_2]	871127-68-3	449.4656
**3**	[(Si**C**SiC)C_1im][$B(CN)_4$]	3027943-07-0	356.3812
**4**	[(Si**C**SiC)C_1im][$N(CN)_2$]	3027943-01-4	307.5421
**6**	[(Si**C**SiC)C_1im][NTf_2]	3051934-11-0	521.6467
**7**	[(Si**O**SiC)C_1im][$B(CN)_4$]	-	358.3540
**8**	[(Si**O**SiC)C_1im][$N(CN)_2$]	-	309.5149
**9**	[(Si**O**SiC)C_1im][NTf_2]	936638-34-5	523.6195

2. Preparation of ionic liquids

Ionic liquids used in this study were prepared following the procedures reported by us previously,[1] and were dried at 10 Pa, 323 K for 24 h prior to use in gas solubility measurements. Water content of the ILs post ethylene solubility measurements was <200 ppm according to coulometric Karl Fischer analysis.

3. MD simulation

3.1 Simulation protocol

Molecular dynamics simulations were performed in LAMMPS software,[2,3] with the USER-DRUDE[4] package activated to treat explicit polarization effects. Initial configurations of periodic cubic boxes were generated using Packmol,[5] and the input files were created with fftool, polarizer and scaleLJ tools.[6,7] A cut-off radius of 12 Å was considered for non-bonded interactions and a tail correction was applied to the van der Waals energy and pressure. The particle-particle particle-mesh (PPPM) method was used to compute long-range electrostatic interactions with an accuracy of $1 \cdot 10^{-5}$. Bonds involving hydrogen atoms were constrained using the SHAKE algorithm. The time step was 1 fs.

Explicit polarization effects were represented by Drude induced dipoles.[8] Each Drude dipole consisted of a positively charged Drude core (DC), placed at the atomic site, and a negatively charged Drude particle (DP) of a mass of 0.4 a.u., connected to the core with a harmonic spring (k_D = 4184 kJ·mol^{-1}). The partial charges of DPs were computed from atomic polarizabilities according to $\alpha = q_D^2/k_D$. Non-hydrogen atoms were considered as polarizable, with the polarizability of neighboring hydrogen atoms merged onto them. The interactions between Drude dipoles were smeared using the Thole function[9,10] with the universal *a* parameter set to 2.6.

The systems were modelled at a temperature of 353 K and a pressure of 0.1 MPa in the NPT ensemble. Drude degrees of freedom were kept at 1 K. The temperature-grouped Nosé-Hoover (tgNH)[11] thermostat and barostat were used for the temperature and pressure control, respectively. When computing free energies, we had to replace the tgNH thermostat with a dual Langevin thermostat due to stability issues during the "activation of a solute in a solvent" step. The NVT ensemble was used to evaluate the intramolecular free energy contribution for the "activation of a solute in vacuum" step.

For the structural analysis, we used simulation boxes consisting of 4 solute molecules placed at the vertices of a tetrahedron with an edge length of 25 Å by 200 ion pair of ILs. The molecules were tethered by a harmonic potential of 5 kcal mol^{-1} to prevent interaction with each other. The systems were equilibrated for 2 ns and then 20 ns production trajectories were generated. The TRAVIS software[12,13] was used to study the local environment of ethylene and acetylene in ionic liquids, and VMD[14] for the visualization.

3.2 Force field development and validation

For modelling ionic liquids, we used the CL&Pol polarizable force field,[15,16] recently extended to Si-based IL cations and to the TCB anion.[17] For C_2H_4 and C_2H_2, a fixed-changed OPLS-AA force field[18] was transformed into a polarizable version. Drude induced dipoles were placed at carbon atoms to represent explicit polarization effects. Atomic polarizabilities of sp^2 and sp carbon atom types were equal to 1.923 and 1.910 Å^3, with the polarizabilities of neighboring hydrogen atoms already taken into account.[19] The non-bonded attractive interactions of a solute with solvent fragments were scaled down to remove double counting of induction effects. The scaling coefficients, evaluated through the predictive scheme, are given in Table S2. The atomic diameters of solute molecules were not modified.

Table S2. Scaling coefficients for the modification of non-bonded attractive interactions.*

Fragment *i*	Fragment *j*	k_{ij}	Fragment *i*	Fragment *j*	k_{ij}
C_2H_2	$C_1C_1im^+$	0.77	C_2H_4	$C_1C_1im^+$	0.80
	TMS	1.00		TMS	1.00
	$(SiC)_2O$	1.00		NTf_2^-	0.69
	NTf_2^-	0.68		$N(CN)_2^-$	0.67
	$N(CN)_2^-$	0.65			
	$B(CN)_4^-$	0.73			

* $C_1C_1im^+$ is a 1,3-dimethylimidazolium fragment, representing the head group of the cations; TMS is a tetramethylsilane fragment, representing the side chain of $[(SiC)C_1im]^+$ and $[(SiCSiC)C_1im]^+$ cations; $(SiC)_2O$ is a hexamethyldisiloxane fragment of $[(SiOSiC)C_1im]^+$.

To validate the obtained polarizable models for ethylene and acetylene, we computed liquid density at boiling point, heat of vaporization of pure ethylene and acetylene. To model the liquid phase, 500 molecules were placed into a cubic periodic simulation box and simulation were carried out at a boiling point (169.0 K for ethylene

and 189.0 K for acetylene) and the pressure at 1 bar in the NPT ensemble. To model the gas phase, 50 molecules were placed into a cubic periodic simulation box at a corresponding gas density (2.09 g·dm^{-3} for ethylene and 1.68 g·dm^{-3} for acetylene), and simulation were carried out in the NVT ensemble. In both cases, 1 ns equilibration and 1 ns production runs were considered.

The enthalpy of vaporization was computed using

$$\Delta H_{vap} = E_g - E_l + RT \qquad \text{(eq. S1)}$$

where E_g and E_l are the total energies per mole in the gas and the liquid phases. The obtained results are given in Table S3. Higher deviations obtained for acetylene can be related to the closeness of melting and boiling points, and therefore difficulty to determine experimentally the liquid properties.

Table S3. MD computed and literature[20–22] liquid densities and heats of vaporization of ethylene and acetylene.

System	ρ_{MD} / g·cm^{-3}	ρ_{lit} / g·cm^{-3}	ΔH_{vap}^{MD} / kJ·mol^{-1}	ΔH_{vap}^{lit} / kJ·mol^{-1}
C_2H_4	0.595	0.526†; 0.568	12.73	13.5 – 14.1
C_2H_2	0.639	0.615	10.87	10.6*; 16.3-17.0§

* calculated value at 273 K; § data in 192-308 K range; †at 170 K and 1.05 bar;

The second validation step included comparison of the solvent structure around the solutes predicted by classical and *ab initio* MD (AIMD) simulations. The systems composed of one solute molecule placed into a cubic periodic box (L ≈ 25.6 – 25.8 Å) containing 30 ion pairs of the smallest ionic liquid, [(SiC)C$_1$im][NTf$_2$], were considered. Prior to the AIMD run, the simulation boxes were equilibrated using classical MD run of 5 ns in the NPT ensemble at 353 K and 1 bar.

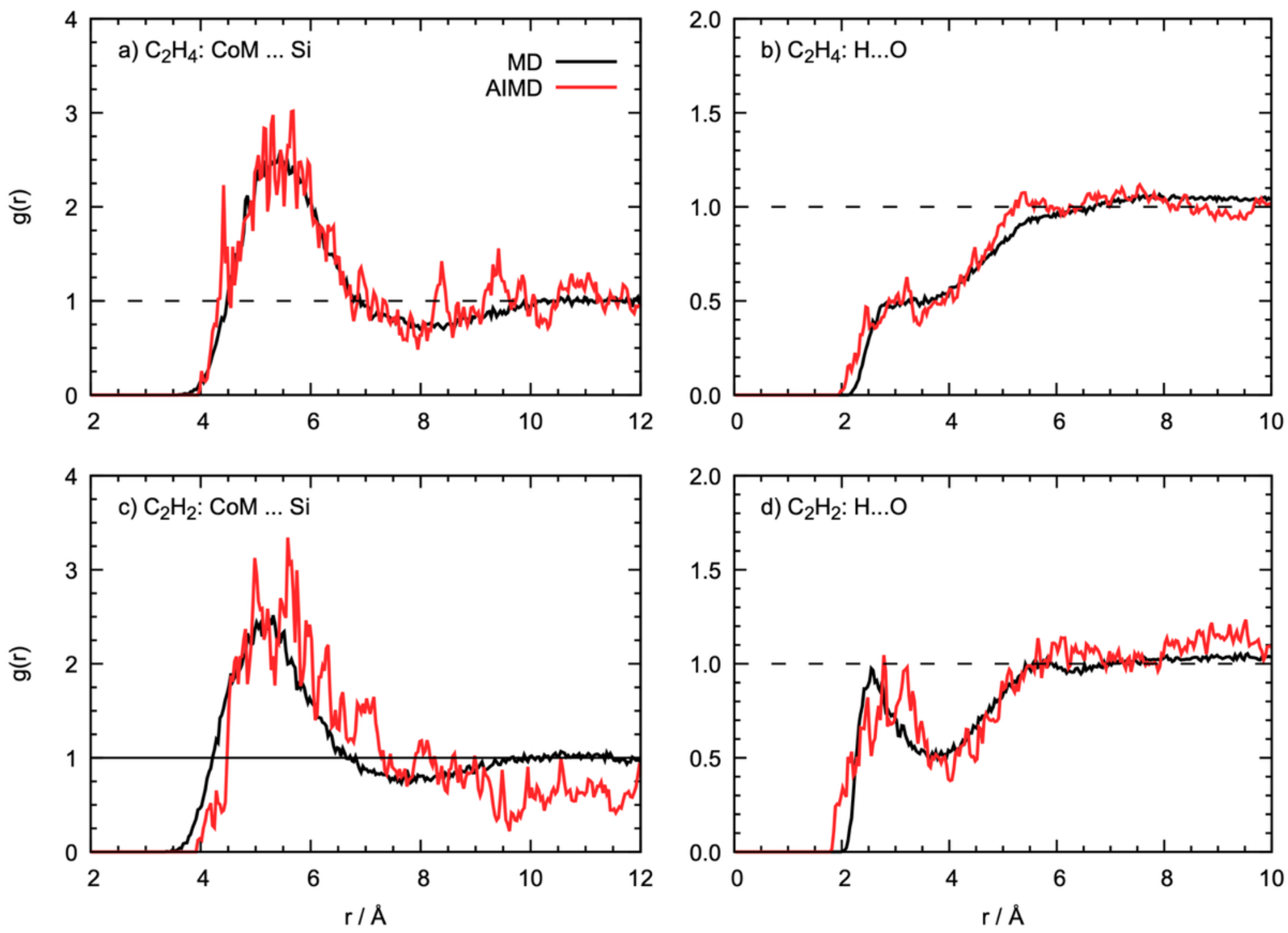

Figure S2. Radial distribution functions, g(r), of Si atoms of the cation (a,c) and oxygen atoms of the anion (b,d) of [(SiC)C_1im][NTf_2] ionic liquid around of centre of mass (CoM) of ethylene (a,b) and acetylene (c,d), obtained from classical (black line) and *ab initio* (red line) MD simulations.

The AIMD simulations at the Density Functional Theory level were carried out using the QUICKSTEP module[23] of the CP2K package.[24] The Perdew-Burke-Ernzerhof (PBE)[25] exchange–correlation functional plus Grimme D3 correction[26] for van der Waals interactions were adopted, with a combination of Gaussian (DZVP-MOLOPT-SR)[27] plus plane waves basis sets (600 Ry) and GTH pseudopotentials.[28] The nuclei displacements were predicted through classical Newton's equations of motions integrated through the velocity-Verlet algorithm, with a timestep of 0.5 fs. Three-dimensional periodic boundary conditions were applied. The simulation was performed for 10 ps in the NVT ensemble at 353 K, using the Nosé-Hoover thermostat with a constant of 0.1 ps to keep the temperature constant. The radial distribution functions were computed using last 8 ps. The corresponding results are given in Figure S2, with the good agreement observed between classical and ab initio MD simulations.

3.3 Free energy calculations

We used the free energy perturbation method (FEP) to compute solvation free energies of ethylene and acetylene in ILs. It was enabled in LAMMPS through the FEP package. Simulation boxes consisted of one freely moving solute molecule surrounded by 100 ion pairs of ILs, with the box length sufficiently large to prevent interaction of a solute with its periodic images.

The free energies were computed using the thermodynamic cycle, which comprises the “activation of a solute in a solvent” and the “activation of a solute in vacuum” independent contributions (Figure S3). During the “activation of a solute in a solvent” step, the interactions were successively turned on in the following order: the van der Waals, electrostatic and induction terms. A coupling parameter λ was increased from λ = 0, when no solute-solvent interactions are present, to λ = 1, when the solute fully interacts with the solvent. The dλ step was 0.05 for Lennard-Jones potential, and 0.1 for Coulomb and polarization terms. At each λ step, a 2 ns equilibration and 2 ns free energy calculations were performed. In order to ensure negligibility of the hysteresis, both “creation’’ (λ+dλ) and ‘‘annihilation’’ (λ-dλ) perturbations of the solute-solvent interactions were considered. Soft-cores potentials were used to avoid singularities at λ = 0. An example of a FEP curve is given in Figure S4. A similar procedure was applied to the “activation of a solute in vacuum” step to estimate the intramolecular term. Since no solvent was present, the simulation times of equilibration and data acquisition were decreased to 100 ps each.

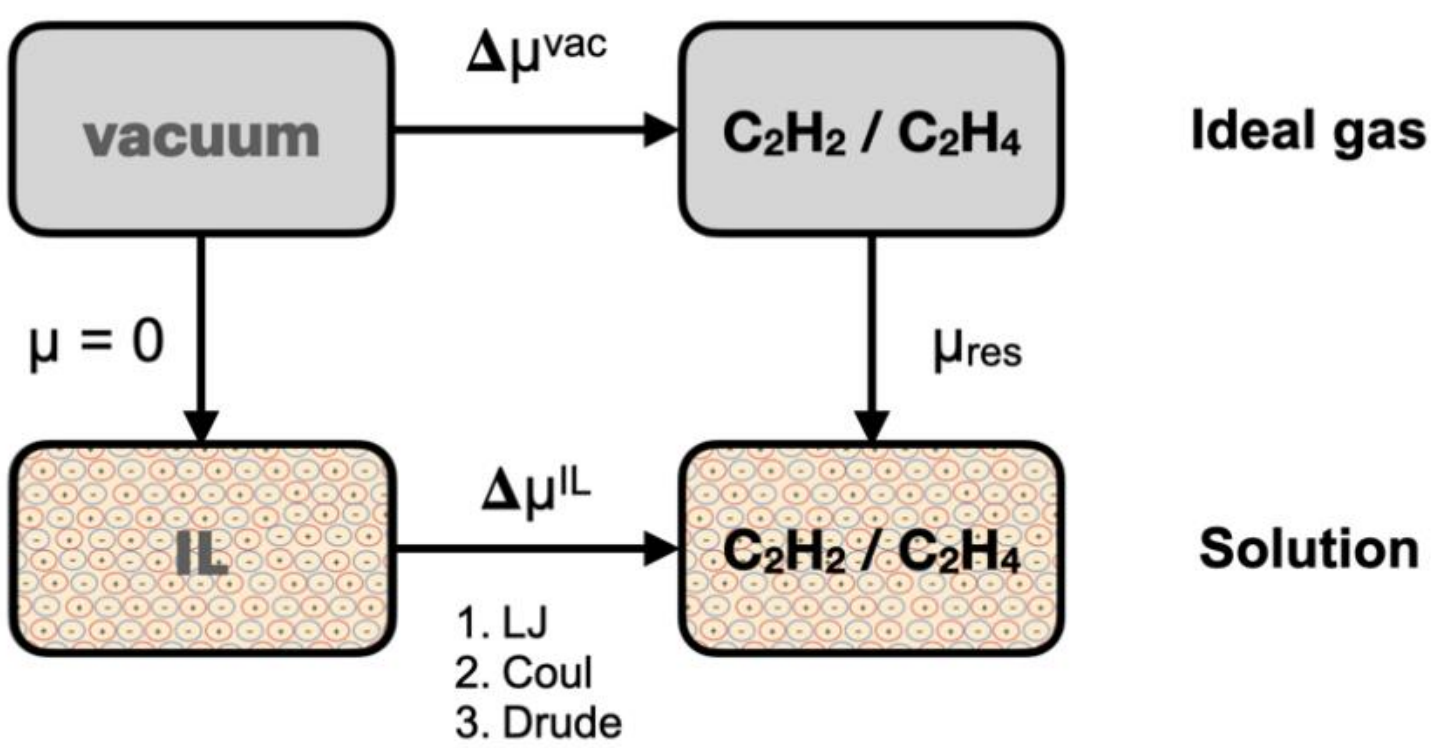

Figure S3. Thermodynamic cycle used to calculate the residual chemical potential.

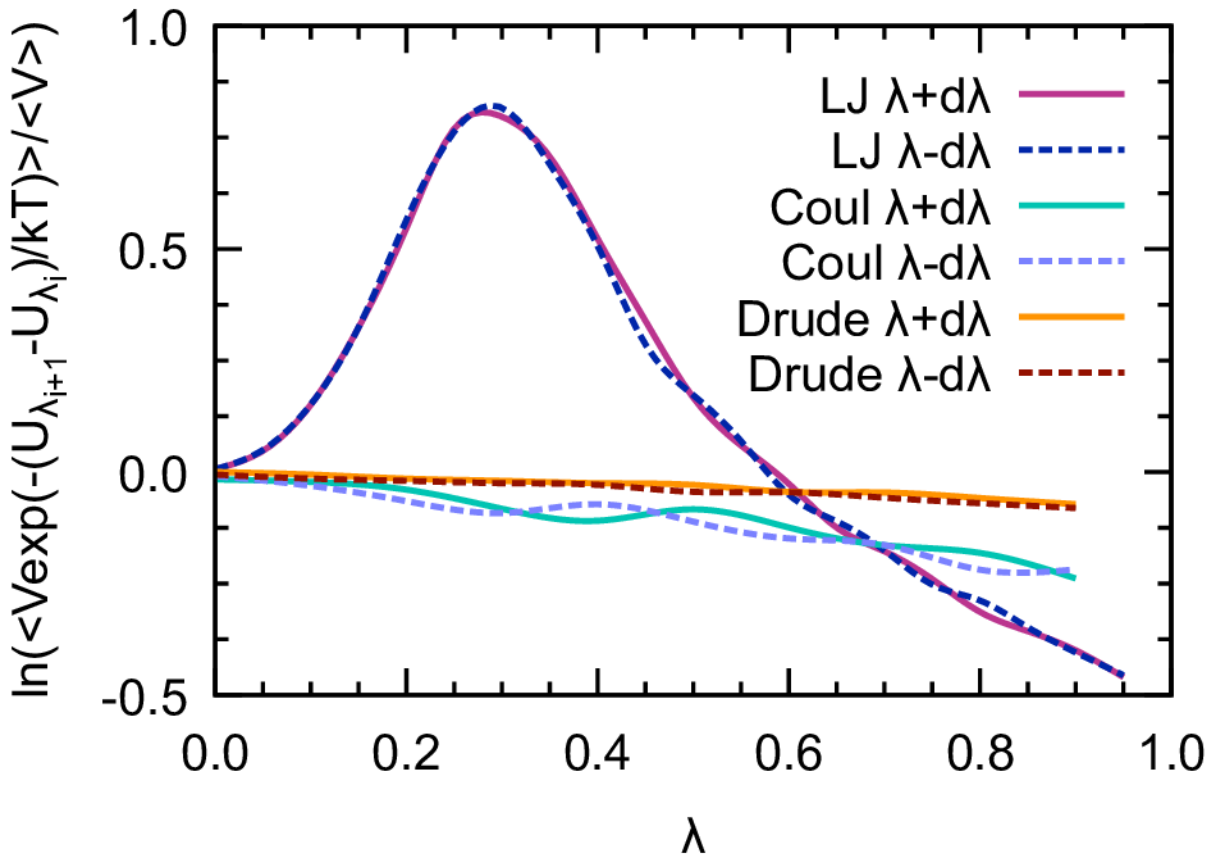

Figure S4. Free energies of "creation" (λ+dλ, solid lines) and "annihilation" (λ-dλ, dashed lines) of C_2H_2 in $[(SiC)C_1im][NTf_2]$ as a function of the activation parameter λ. LJ corresponds to the activation of Lennard-Jones term, Coul to Coulomb term, and Drude to polarization term. For all the terms, the intramolecular (vacuum) contribution is subtracted.

The chemical potential difference between the initial and the final states in the NPT ensemble was evaluated through the relation

$$\Delta\mu_i = -k_B T \ln \frac{\langle V \exp(-\Delta U / k_B T) \rangle_{\lambda_i}}{\langle V \rangle_{\lambda_i}} \qquad \text{(eq. S2)}$$

Then, the residual chemical potential of the whole thermodynamic cycle was computed as

$$\mu_{res} = \sum_i \Delta\mu_i \qquad \text{(eq. S3)}$$

where *i* corresponded to the van der Waals, electrostatic and induction terms. From this value, the mole fraction solubility of ethylene or acetylene in an IL was computed

$$x = \frac{p^*}{\rho_{IL} k_B T} \exp\left(\frac{\mu_{res}}{k_B T}\right) \qquad \text{(eq. S4)}$$

where ρ_{IL} is the molar density of the IL solvent and p^* is the partial pressure of the gas (1 bar).

3.4 Simulation results

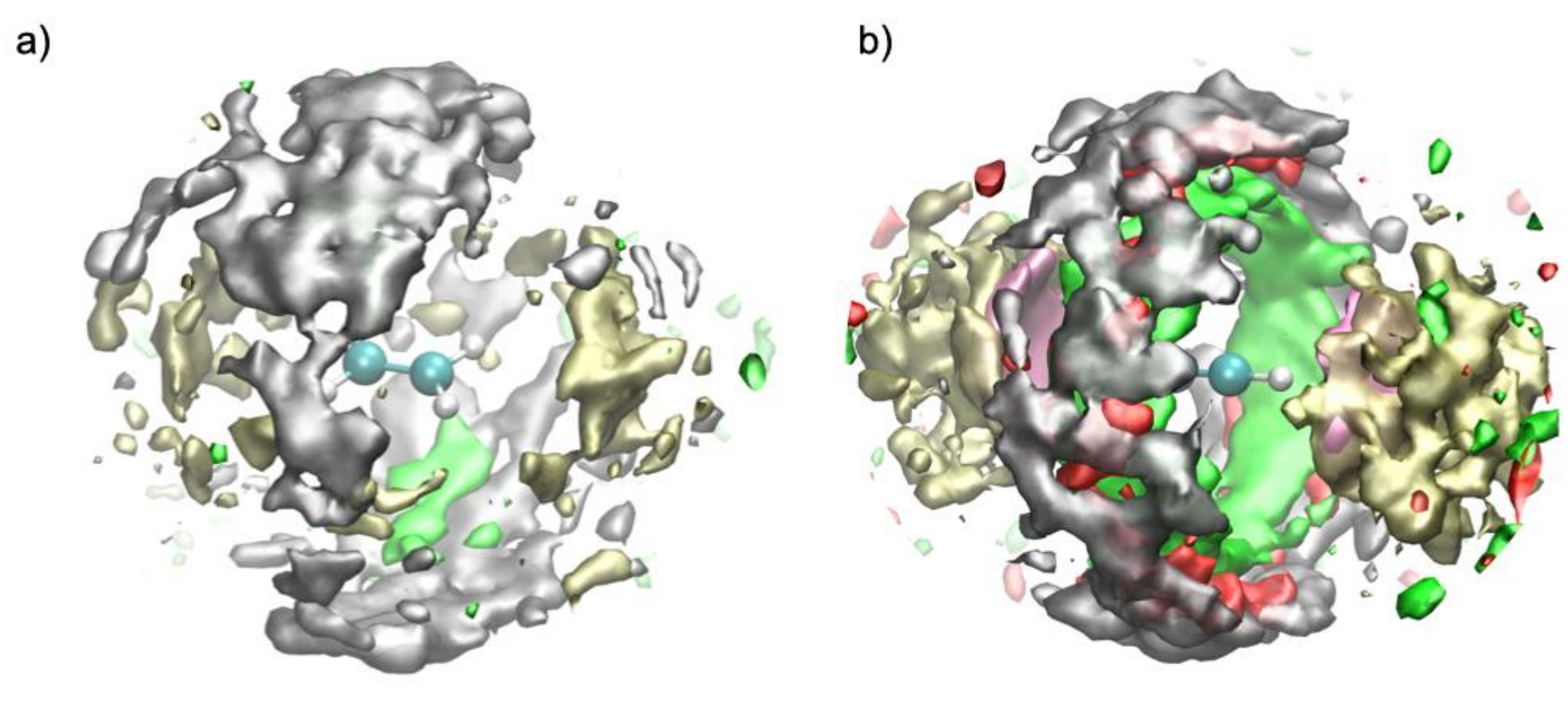

Figure S5. Spatial distribution functions illustrating the local environment of ethylene (a) and acetylene (b) in [(SiOSiC)C_1im][NTf_2] ionic liquid. The grey surface corresponds to Si atoms, green to CH_3-N carbon atoms, red to centre of imidazolium ring (CoR) of cations, tan to F atoms, pink to O atoms of anions (isodensity contours at 3.3, 2.0, 2.0, 0.26 and 0.50 times the average density, respectively)

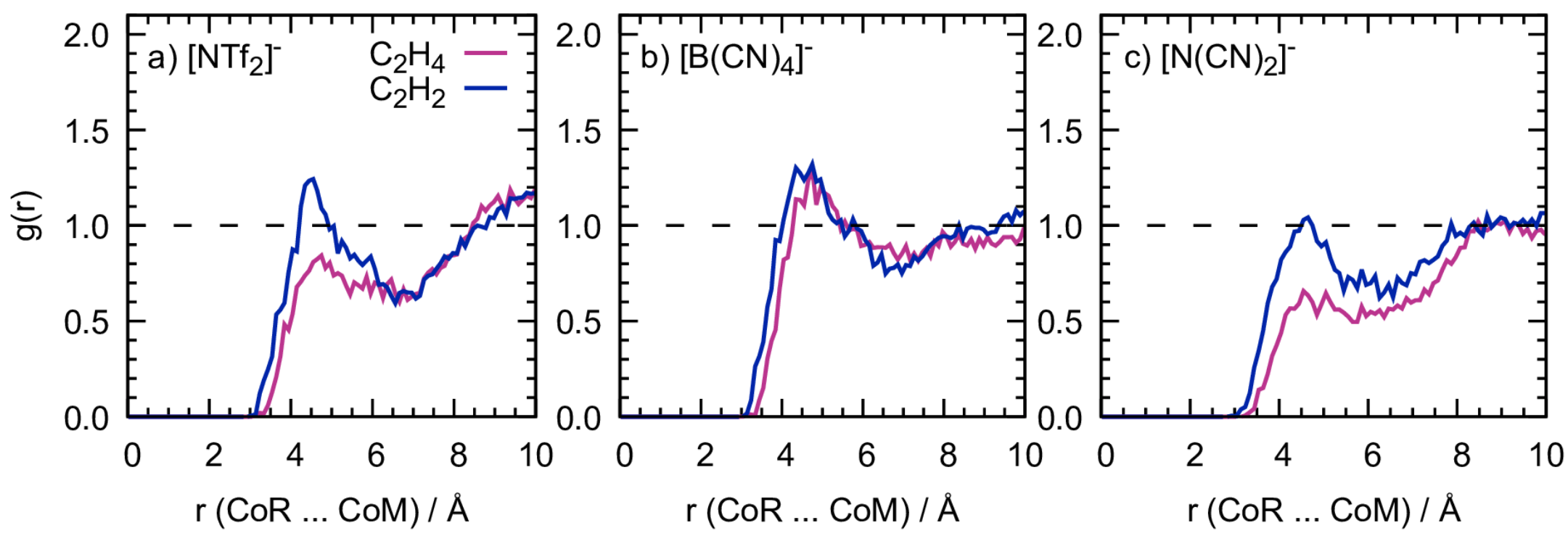

Figure S6. Radial distribution functions, g(r), of centre of mass (CoM) of ethylene (rose line) and acetylene (blue line) around centre of ring (CoR) of imidazolium head group in [(SiCSiC)C_1im][NTf_2] (a), [(SiCSiC)C_1im][$B(CN)_4$] (b) and [(SiCSiC)C_1im][$N(CN)_2$] (c) ionic liquids.

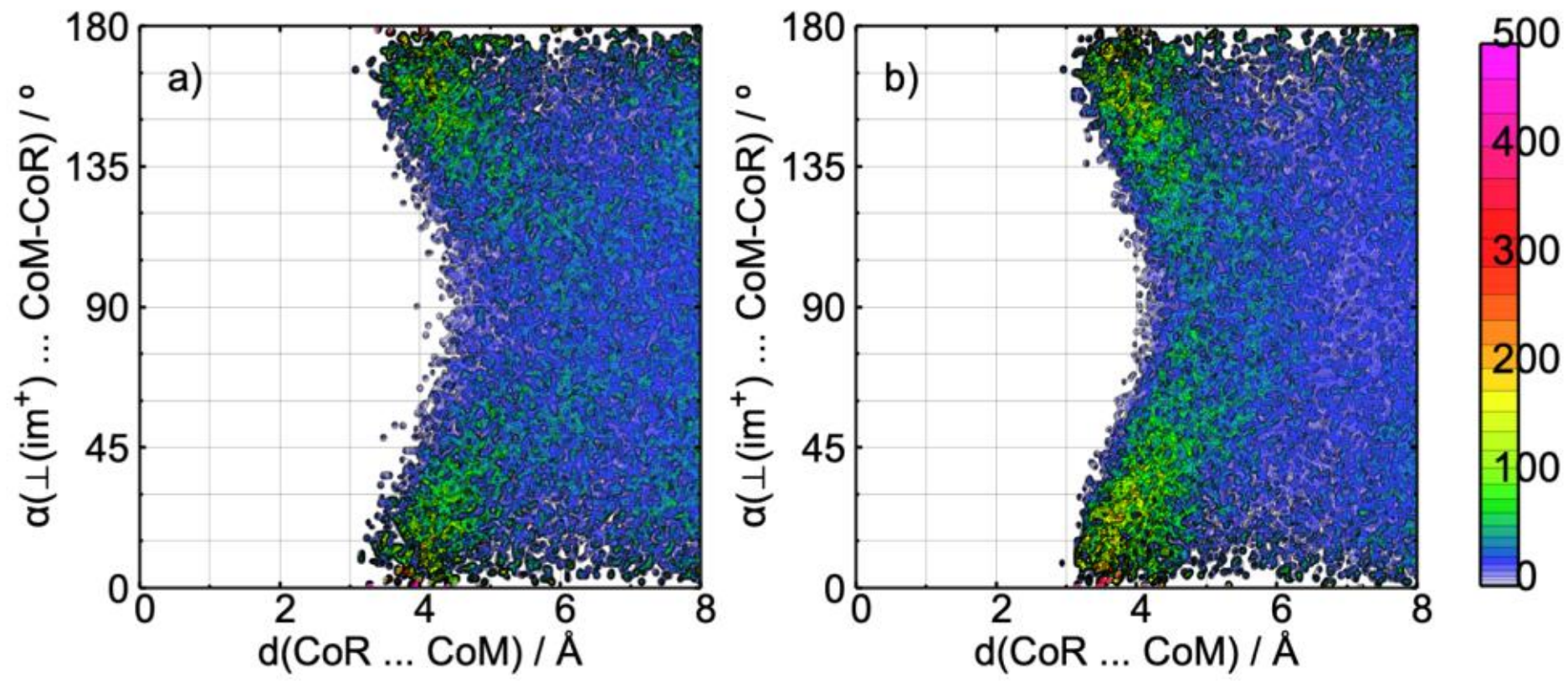

Figure S7. Probability contours revealing π-stacking between ethylene (a) and acetylene (b) and imidazolium head group of [(SiOSiC)C_1im][NTf_2] ionic liquid. The *x*-axis represents the distance between the centre of mass (CoM) of the solute and the centre of ring (CoR) of imidazolium ring. The *y*-axis represents the angle formed by the normal to imidazolium plane (⊥(im$^+$)) and the CoM-CoR vector.

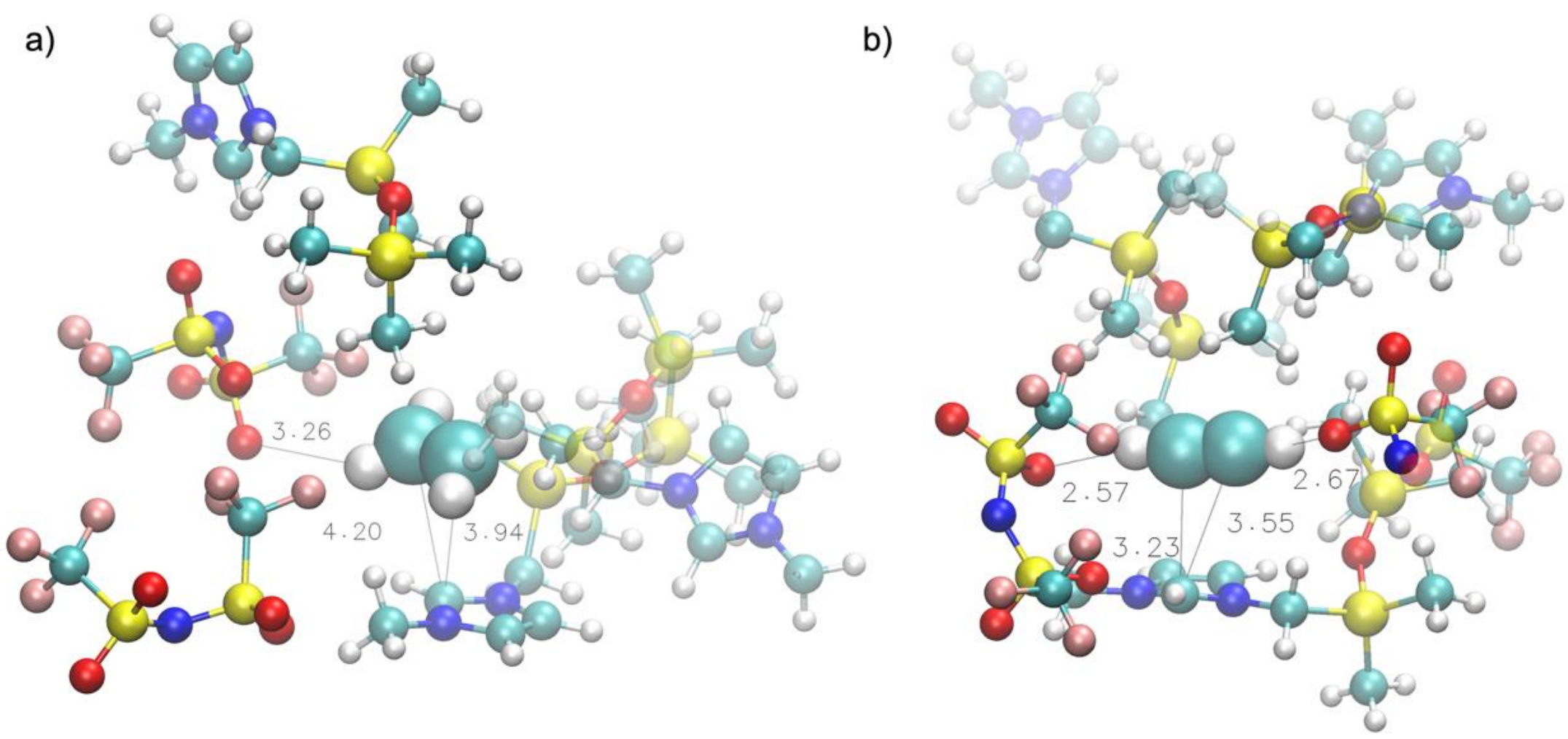

Figure S8. Simulation snapshot illustrating solvation environment of ethylene (a) and acetylene (b) in [(SiOSiC)C_1im][NTf_2] ionic liquid.

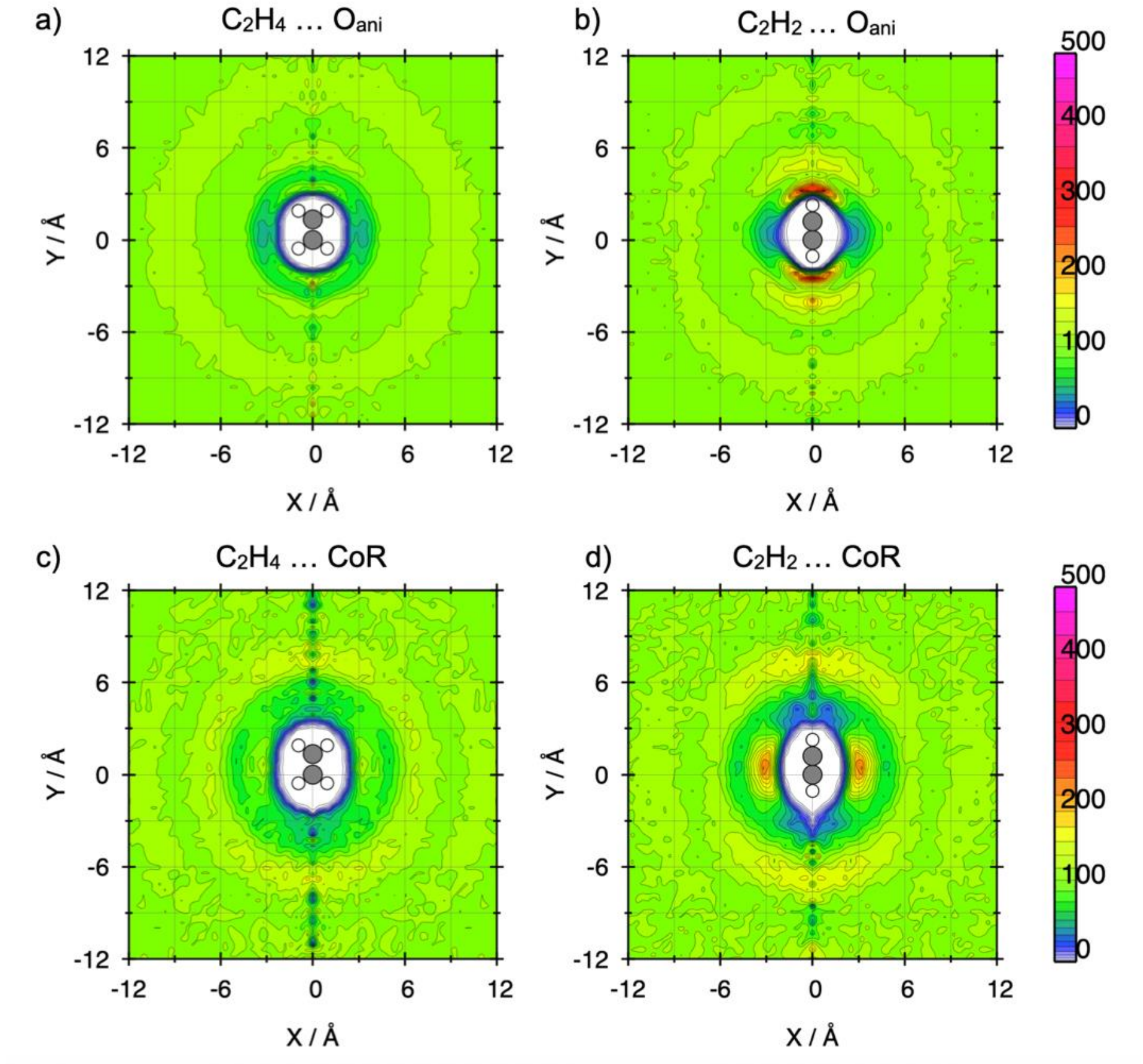

Figure S9. Pseudo-spatial distribution functions illustrating the local environment of ethylene (a,c) and acetylene (b,d) in [(SiOSiC)C_1im][NTf_2] ionic liquid. Two carbon atoms of the solutes are chosen as reference atoms. Schematic representation of the orientation of ethylene or acetylene molecule is given at the centre of each sub-figure. Top sub-figures (a,b) refer to the oxygen atoms of the [NTf_2]$^-$ anion, and bottom (c,d) to centre of imidazolium ring (CoR) of [(SiOSiC)C_1im]$^+$ cation.

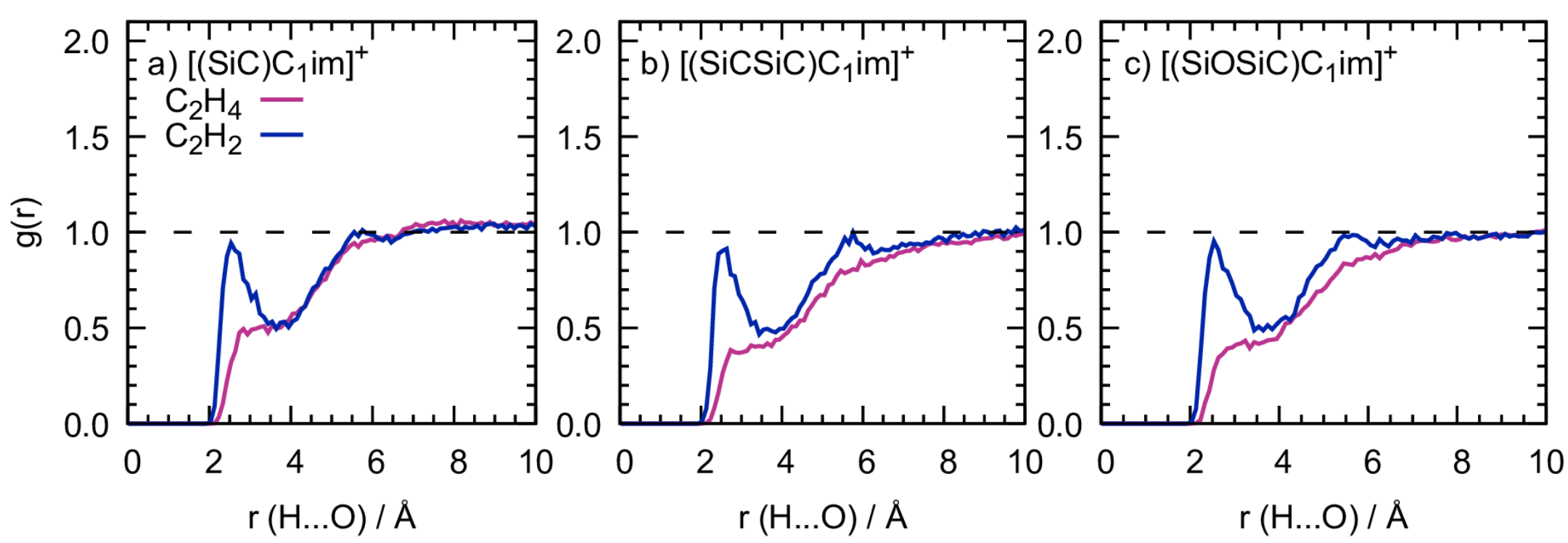

Figure S10. Radial distribution functions, g(r), of hydrogen atoms of ethylene (rose line) and acetylene (blue line) around oxygen atoms of the [NTf_2]$^-$ anion in [(SiC)C_1im][NTf_2] (a), [(SiCSiC)C_1im][NTf_2] (b) and [(SiOSiC)C_1im][NTf_2] (c).

4. Gas solubility measurements

In previous publications,[1,29,30] custom-built glass apparatuses were employed for volumetric isochoric saturation measurements involving various gas–liquid systems. However, after a recent miniaturization of the glass cell design, a small yet non-negligible absorption of hydrocarbon gases was observed in the lubricant used for the glass joints.[31]

To eliminate potential systematic deviations arising from such absorption, a newly designed, all-metal, lubricant-free equilibrium cell was developed and used in the present ethylene solubility measurements. This cell is described in the section 4.1. The study of the solubility of ethylene in different ionic liquids, in the temperature interval between 303-338 K and up to 0.14 MPa is presented in section 4.2.

4.1 Description of the new metallic cell

Although the general configuration of the setup remains consistent with previously reported assemblies, the current version of the cell is constructed entirely from stainless steel and is schematically represented in Figure S11.

The metallic system comprises an equilibrium cell immersed in a thermostatic bath (model CC-130A, Huber), with temperature regulation provided by a PID controller. The temperature was measured using a platinum resistance thermometer (PT100) connected to a Hart Scientific readout (model 1502A), calibrated between 77 K and 693 K, with an accuracy of ± 0.010 K at 273 K and a resolution of 0.001 K.

The cell is connected to a glass manifold through a Rotulex metal–glass joint. This connection allows the entire system to be evacuated, returned to atmospheric pressure, or charged with gas (in this work, ethylene) as required for the experimental procedure. For that, the glass line is connected with a vacuum pump (Edwards RV3, capable of maintaining pressures as low as 4 Pa) and a gas and air inlet system. The pressure inside the cell is monitored using a manometer (model RPS/DPS 800, Druck, GE) based on resonant silicon pressure sensor technology. The instrument covers a measurement range from 0.0035 MPa to 0.262 MPa, with an accuracy of ± 0.01% of full scale. The pressure transducer is thermostated at the same temperature as the water bath to ensure a homogeneous temperature and avoid pressure gradients. The constant internal volume of the metallic cell is maintained using Swagelok stainless-steel ball valves. If the cell

is disassembled, it must be reassembled with all components positioned exactly as in the original configuration, using a reference positioning tool to reproduce the same geometry as closely as possible. Nevertheless, to ensure accuracy, the internal volume of the cell should be recalibrated after each reassembly.

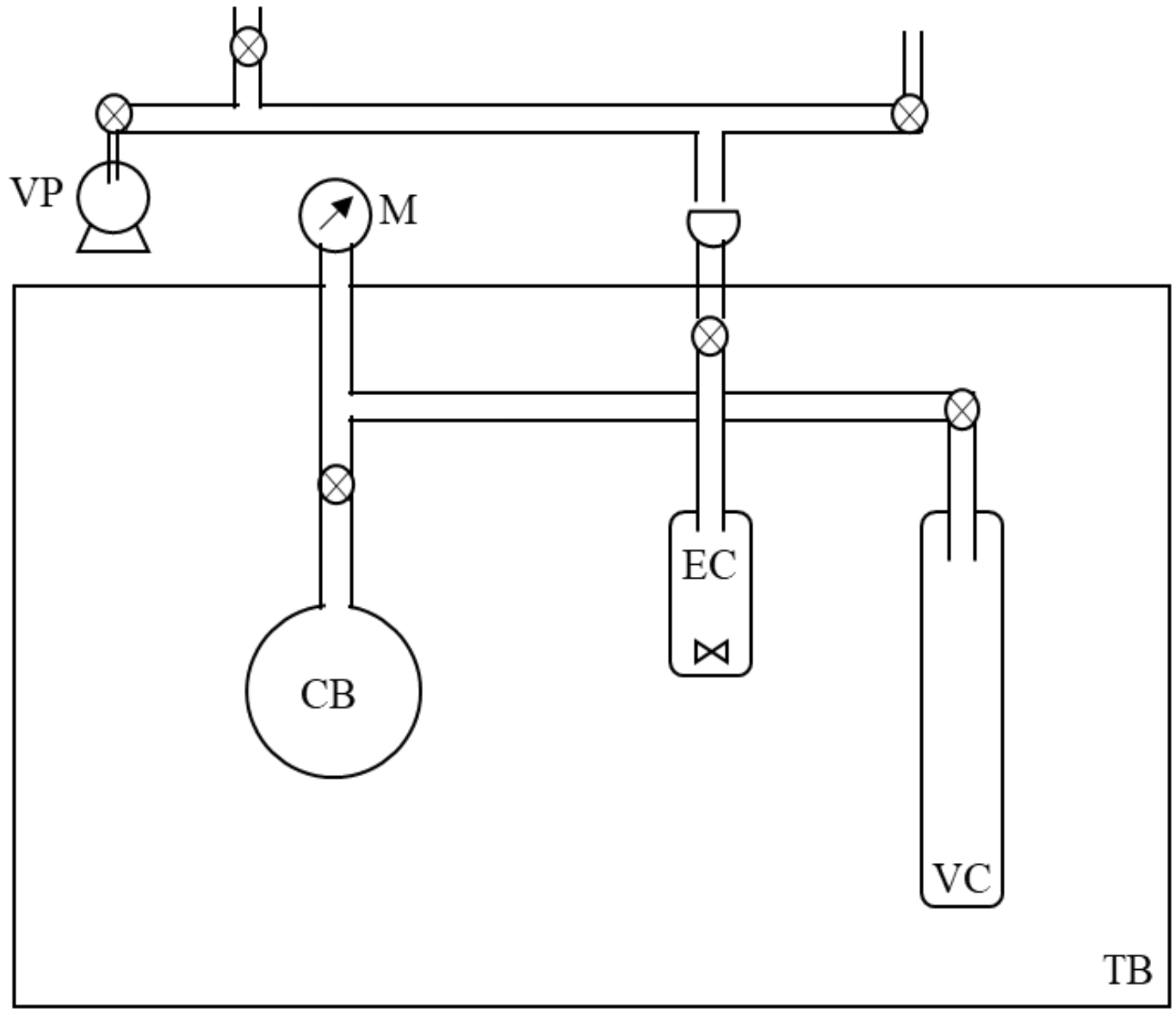

Figure S11. Schematic representation of the solubility apparatus: VP, vacuum pump; VC, vapour phase chamber; CB, calibration bulb; M, precise manometer; EC, equilibrium chamber; TB, thermostated bath.

4.2 Assumptions adopted in the solubility calculations

4.2.1 Negligible vapor pressure of the ionic liquids

In the gas solubility results presented in Section 4.3, the vapor pressure of the ionic liquids was assumed to be negligible relative to the equilibrium pressure measured in the gas phase. This assumption is justified by previously reported vapor pressure measurements for the studied ionic liquids.[32] As reported previously, the vapor pressures of the ionic liquids are extremely low, with values ranging from approximately 10^{-2} to 10^{-1} Pa over the temperature interval 443–503 K. Since the gas solubility measurements reported in this work were performed at lower temperatures

(303–338 K), the vapor pressures of the ionic liquids under the experimental conditions are expected to be even lower than those reported.

The equilibrium pressures measured during the ethylene absorption experiments are on the order of 0.1 MPa. Therefore, the contribution of ionic liquid vapor to the total pressure in the equilibrium cell is negligible and the measured equilibrium pressure can be assumed to correspond exclusively to ethylene.

4.2.2 Negligible liquid volume change upon gas absorption

The volume of the IL was assumed not to change upon gas absorption. This approximation is justified by the relatively low amount of ethylene absorbed under the experimental conditions investigated. Neglecting the swelling of the ionic liquid upon gas absorption is expected to introduce a negligible contribution to the uncertainty of the calculated solubility as demonstrated below.

The liquid expansion was estimated using the highest solubility measured in this work ($x_2 = 25.49 \cdot 10^{-3}$, $T = 303.44$ K, IL [(SiCSiC)C_1im][NTf_2]), according to:

$$\Delta V = V_{IL,g} - V_{IL,0}$$

where $V_{IL,0}$ is the volume of the pure IL before absorption:

$$V_{IL,0} = x_{IL} V^*_{IL}$$

For a basis of 1 mol of IL: $V_{IL,0} = 402.7$ $cm^3 \cdot mol^{-1}$

This molar volume was obtained from the density of the IL at 303 K ($\rho = 1.2954$ $g \cdot cm^{-3}$) and its molar mass (521.6467 $g \cdot mol^{-1}$)[1]

The volume of the liquid phase after gas absorption is calculated as:

$$V_{IL,g} = x_{IL} V^*_{IL} + x_g V_g$$

where V_L and V_g are the partial molar volumes of the ionic liquid and dissolved ethylene, respectively. Assuming that the partial molar volume of the IL is not significantly affected by the presence of the small amount of dissolved ethylene ($V_L \approx V_{L,0}$) then:

$$\Delta V = n_L V_L + n_g V_g - V_{L,0}$$

Is similar to:

$$\Delta V = n_g V_g$$

For the highest measured solubility, the absorbed amount corresponds approximately to 0.026 mol of ethylene per mol of IL ($n_g = x_2/(1-x_2)$)

Based on literature values[33] for the partial molar volume of ethylene at infinite dilution in non-polar organic liquids at 298 K, V_g can be estimated as approximately 58 $cm^3 \cdot mol^{-1}$. This results in an estimated liquid expansion of 1.57 $cm^3 \cdot mol^{-1}$, corresponding to only 0.38% of the initial IL volume. Thus, even under the maximum ethylene uptake measured in this work, the estimated liquid expansion is below 0.4% of the initial liquid volume and was therefore neglected in the solubility calculations.

4.3. Gas solubility results

The experimental data points regarding the solubility results are summarized in table S4 as Henry's law constants (K_H / Pa) and as mole fraction of the solute (x_2). The molar fraction of the solute was calculated from the experimental data on K_H, assuming a partial pressure of gas equal to 0.1 MPa. This corresponds to expressing the solubility data at a common reference pressure for comparison between ionic liquids. As the experimental equilibrium pressures are close to 0.1 MPa, the uncertainty introduced by this normalization is negligible.

The raw experimental solubility data was fitted as a function of temperature (T / K) by an empirical equation of the type:

$$\ln\left[K_H(T)(10^5\,\mathrm{Pa})\right] = \sum_{i=0}^{n} A_i (T(\mathrm{K}))^{-i} \qquad \text{(eq. S5)}$$

the coefficients A_i obtained are listed in table S5 together with the average relative deviations obtained for each IL solvent. These values give an overview of the precision of the experimental data which is in the present case always better than 1.2 %. The experimental solubility (expressed as molar fraction) is represented in Figure S12 as a function of the experimental temperatures studied.

Table S4. Experimental values obtained for the solubility of ethylene in different pure ionic liquids, expressed both as Henry's law constants, K_H, and as ethylene mole fraction[a], x_2.

T / K	p [b] / 10^2 Pa	$x_{2,exp}$ [a] $\cdot 10^3$	K_H / 10^5 Pa	$x_{2,\,0.1\,MPa}$ [a] $\cdot 10^3$	Dev. [c] / %
		**[(Np)C$_1$im][NTf$_2$]**			
303.45	1320.8	16.78	78.14	12.730	+0.06
308.43	1343.7	15.99	83.47	11.920	-0.06
313.40	1292.2	14.418	89.06	11.174	-0.06
318.39	1389.3	14.55	94.85	10.494	-0.08
323.35	1335.1	13.158	100.87	9.870	-0.01
328.36	1434.7	13.28	107.4	9.27	+0.3
333.30	1377.7	12.094	113.30	8.791	-0.05
338.27	1398.9	11.62	119.7	8.321	-0.1
		**[(SiC)C$_1$im][NTf$_2$]**			
303.45	1319.7	18.6	70.3	14.15	+0.6
308.43	1343.5	17.8	75.1	13.26	+0.6
308.43	1244.4	16.63	74.4	13.38	-0.4
310.91	1178.3	15.14	77.4	12.86	+0.4
313.41	986.9	12.5	78.7	12.6	-1
313.42	1265.7	15.88	79.2	12.56	-0.4
318.38	1207.4	14.13	85.0	11.71	+0.5
318.39	870.3	10.31	84.1	11.84	-0.6
318.39	1286.8	15.13	84.6	11.77	-0.09
318.39	1391.1	16.2	85.4	11.7	+0.9
323.36	884.2	9.86	89.3	11.15	-0.6
323.37	1019.9	11.37	89.3	11.15	-0.6
328.35	1438.1	14.9	96.1	10.36	+0.9
328.36	1328.9	13.877	95.23	10.457	+0.00
333.34	1052.6	10.47	100.1	9.95	-0.6
338.30	925.9	8.62	107.1	9.30	+0.6
		**[(SiCSiC)C$_1$im][NTf$_2$]**			
303.44	1297.2	25.49	50.54	19.682	+0.02
308.43	1173.5	21.4	54.6	18.2	+0.8

T / K	p [b] / 10^2 Pa	$x_{2,exp}$ [a] $\cdot 10^3$	K_H / 10^5 Pa	$x_{2,\,0.1\,MPa}$ [a] $\cdot 10^3$	Dev. [c] / %
313.38	1343.8	23.1	57.7	17.24	-0.5
318.39	1212.8	19.3	62.4	16.0	+0.7
318.56	1367.8	22.0	61.7	16.1	-0.7
323.32	1389.7	21.2	65.2	15.3	-1
328.36	1251.9	17.64	70.6	14.10	+0.5
338.33	1290.7	16.19	79.4	12.55	+0.4
		**[(SiCSiC)C_1im][N$(CN)_2$]**			
303.45	1321.7	14.49	90.6	10.98	+0.1
308.42	1311.5	13.54	96.2	10.34	-0.3
313.40	1367.3	13.13	103.4	9.62	+0.7
318.38	1356.0	12.5	108	9.2	-1
323.36	1412.7	12.0	117	8.5	+1
328.34	1400.7	11.5	121	8.21	-1
333.31	1457.7	11.0	132	7.6	+2
338.31	1445.1	10.60	136	7.35	-0.9
		**[(SiCSiC)C_1im][B$(CN)_4$]**			
303.45	1271.0	21.46	58.8	16.91	-0.3
308.42	1232.0	19.3	63.3	15.71	+0.6
313.40	1316.1	19.56	66.9	14.88	-0.3
318.38	1274.6	17.73	71.5	13.93	+0.2
323.35	1360.9	17.90	75.6	13.18	-0.1
328.33	1316.9	16.36	80.1	12.436	-0.07
333.28	1405.3	16.493	84.73	11.755	+0.04
		**[(SiOSiC)C_1im][B$(CN)_4$]**			
303.45	917.5	17.3	52.7	18.9	-1
308.42	1293.7	22.2	57.9	17.2	+1
313.39	949.4	15.7	60.4	16.5	-1
318.37	1339.1	20.2	65.9	15.1	+1
323.33	980.9	14.2	68.6	14.5	-0.7
328.32	1384.1	18.5	74.3	13.4	+1
333.27	1012.1	13.1	76.9	13.0	-1
		**[(SiOSiC)C_1im][N$(CN)_2$]***			
308.42	1055.6	11.663	90.03	11.051	-0.07
313.39	1073.9	11.122	96.05	10.361	-0.03
318.36	1092.1	10.62	102.36	9.725	+0.09
328.31	1128.2	9.74	115.3	8.63	+0.1
338.23	1164.0	9.01	128.7	7.743	-0.1
		**[(SiOSiC)C_1im][NTf_2]**			
303.45	1155.0	23.1	49.7	20.0	-0.7
303.49	1104.5	21.95	50.02	19.88	-0.06
308.41	1175.0	22.1	52.8	18.8	-0.6
308.43	1123.5	21.044	53.09	18.742	-0.01
308.43	1116.8	20.8	53.5	18.6	+0.8

T / K	p [b] / 10^2 Pa	$x_{2,exp}$ [a] $\cdot 10^3$	K_H / 10^5 Pa	$x_{2,\,0.1\,MPa}$ [a] $\cdot 10^3$	Dev. [c] / %
313.40	1195.0	21.2	56.0	17.79	-0.5
313.41	1142.7	20.17	56.3	17.67	+0.2
313.41	1142.7	20.13	56.5	17.63	+0.4
318.38	1161.7	19.38	59.6	16.70	+0.3
318.40	1154.7	19.2	59.9	16.6	+0.7
323.35	1234.8	19.7	62.3	16.0	-0.7
323.36	1180.8	18.66	62.9	15.82	+0.3
328.33	1199.7	18.00	66.3	15.01	+0.3
328.36	1192.3	17.89	66.3	15.02	+0.2
333.31	1218.6	17.45	69.5	14.33	-0.1
338.256	1237.4	16.81	73.3	13.60	+0.3
338.318	1229.8	16.9	72.5	13.7	-0.8

[a] $x_{2,\,exp}$ is the ethylene molar fraction obtained in equilibrium at the experimental conditions; $x_{2,\,0.1MPa}$ is the ethylene molar fraction corrected for a partial pressure of solute of 0.1MPa; [b] p is the experimental equilibrium pressure; [c] Dev. is the percent deviation of the experimental data relatively to the correlations reported in Table S2.

* Unlike in all other ILs, ethylene absorption measurements in [(SiOSiC)C_1im][$N(CN)_2$] were done using a single IL mass (filling), therefore the uncertainties for ethylene solubility might be underestimated.

Table S5. Parameters of eq. S1 used to fit the experimental results presented in The **study of the solubility of ethylene in different ionic liquids, in the temperature interval between 303-338 K and up to 0.14 MPa is presented in section 4.2.**

4.1 Description of the new metallic cell

Although the general configuration of the setup remains consistent with previously reported assemblies, the current version of the cell is constructed entirely from stainless steel and is schematically represented in Figure S11.

The metallic system comprises an equilibrium cell immersed in a thermostatic bath (model CC-130A, Huber), with temperature regulation provided by a PID controller. The temperature was measured using a platinum resistance thermometer (PT100) connected to a Hart Scientific readout (model 1502A), calibrated between 77 K and 693 K, with an accuracy of ± 0.010 K at 273 K and a resolution of 0.001 K.

The cell is connected to a glass manifold through a Rotulex metal–glass joint. This connection allows the entire system to be evacuated, returned to atmospheric pressure, or charged with gas (in this work, ethylene) as required for the experimental procedure. For that, the glass line is connected with a vacuum pump (Edwards RV3, capable of maintaining pressures as low as 4 Pa) and a gas and air inlet system. The pressure inside the cell is monitored using a manometer (model RPS/DPS 800, Druck, GE) based on resonant silicon pressure sensor technology. The instrument covers a measurement range from 0.0035 MPa to 0.262 MPa, with an accuracy of ± 0.01% of full scale. The pressure transducer is thermostated at the same temperature as the water bath to ensure a homogeneous temperature and avoid pressure gradients. The constant internal volume of the metallic cell is maintained using Swagelok stainless-steel ball valves. If the cell is disassembled, it must be reassembled with all components positioned exactly as in the original configuration, using a reference positioning tool to reproduce the same geometry as closely as possible. Nevertheless, to ensure accuracy, the internal volume of the cell should be recalibrated after each reassembly.

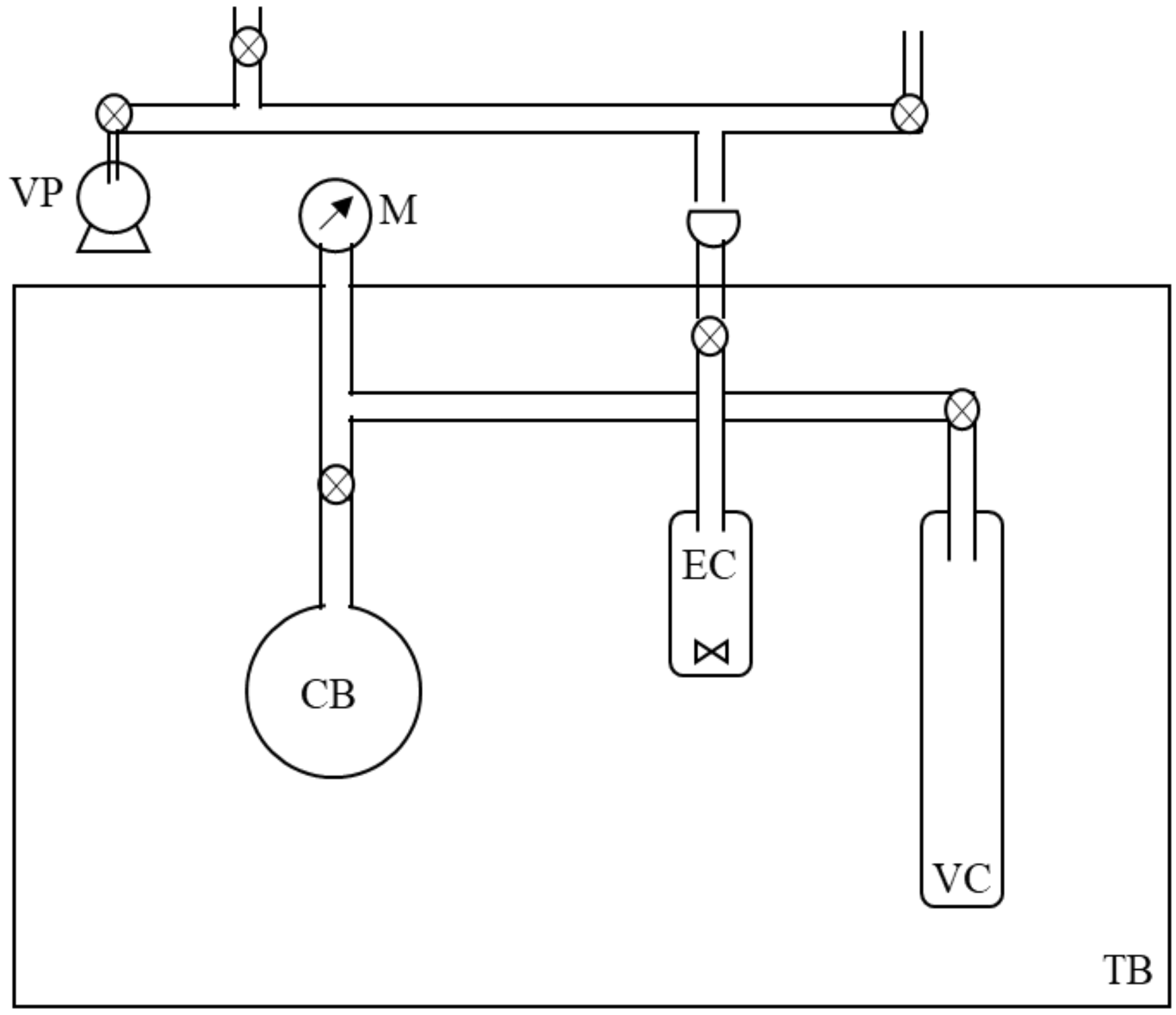

Figure S11. Schematic representation of the solubility apparatus: VP, vacuum pump; VC, vapour phase chamber; CB, calibration bulb; M, precise manometer; EC, equilibrium chamber; TB, thermostated bath.

4.2 Assumptions adopted in the solubility calculations

4.2.1 Negligible vapor pressure of the ionic liquids

In the gas solubility results presented in Section 4.3, the vapor pressure of the ionic liquids was assumed to be negligible relative to the equilibrium pressure measured in the gas phase. This assumption is justified by previously reported vapor pressure measurements for the studied ionic liquids.[32] As reported previously, the vapor pressures of the ionic liquids are extremely low, with values ranging from approximately 10^{-2} to 10^{-1} Pa over the temperature interval 443–503 K. Since the gas solubility measurements reported in this work were performed at lower temperatures (303–338 K), the vapor pressures of the ionic liquids under the experimental conditions are expected to be even lower than those reported.

The equilibrium pressures measured during the ethylene absorption experiments are on the order of 0.1 MPa. Therefore, the contribution of ionic liquid vapor to the total

pressure in the equilibrium cell is negligible and the measured equilibrium pressure can be assumed to correspond exclusively to ethylene.

4.2.2 Negligible liquid volume change upon gas absorption

The volume of the IL was assumed not to change upon gas absorption. This approximation is justified by the relatively low amount of ethylene absorbed under the experimental conditions investigated. Neglecting the swelling of the ionic liquid upon gas absorption is expected to introduce a negligible contribution to the uncertainty of the calculated solubility as demonstrated below.

The liquid expansion was estimated using the highest solubility measured in this work (x_2 = 25.49·10[-3], T = 303.44 K, IL [(SiCSiC)C_1im][NTf_2]), according to:

$$\Delta V = V_{L,g} - V_{L,0}$$

where $V_{L,0}$ is the volume of the pure IL before absorption:

$$V_{L,0} = x_L V_L^*$$

For a basis of 1 mol of IL: $V_{L,0}$ = 402.7 cm^3·mol^{-1}

This molar volume was obtained from the density of the IL at 303 K (ρ = 1.2954 g·cm^{-3}) and its molar mass (521.6467 g·mol^{-1})[1]

The volume of the liquid phase after gas absorption is calculated as:

$$V_{L,g} = x_L \bar{V}_L + x_g \bar{V}_g$$

where $\bar{V}_L$ and $\bar{V}_g$ are the partial molar volumes of the ionic liquid and dissolved ethylene, respectively. Assuming that the partial molar volume of the IL is not significantly affected by the presence of the small amount of dissolved ethylene ($\bar{V}_L \approx V_L^*$), then:

$$\Delta V = x_L \bar{V}_L + x_g \bar{V}_g - V_{L,0}$$

Is similar to:

$$\Delta V_L = n_g \bar{V}_g$$

For the highest measured solubility, the absorbed amount corresponds approximately to 0.026 mol of ethylene per mol of IL ($n_g = x_2/(1-x_2)$)

Based on literature values[33] for the partial molar volume of ethylene at infinite dilution in non-polar organic liquids at 298 K, $\bar{V}_g$ can be estimated as approximately 58 $cm^3 \cdot mol^{-1}$. This results in an estimated liquid expansion of 1.57 $cm^3 \cdot mol^{-1}$, corresponding to only 0.38% of the initial IL volume. Thus, even under the maximum ethylene uptake measured in this work, the estimated liquid expansion is below 0.4% of the initial liquid volume and was therefore neglected in the solubility calculations.

4.3. Gas solubility results

The experimental data points regarding the solubility results are summarized in table S4 as Henry's law constants (K_H / Pa) and as mole fraction of the solute (x_2). The molar fraction of the solute was calculated from the experimental data on K_H, assuming a partial pressure of gas equal to 0.1 MPa. This corresponds to expressing the solubility data at a common reference pressure for comparison between ionic liquids. As the experimental equilibrium pressures are close to 0.1 MPa, the uncertainty introduced by this normalization is negligible.

The raw experimental solubility data was fitted as a function of temperature (T / K) by an empirical equation of the type:

$$\ln\left[K_H(T)(10^5\,\mathrm{Pa})\right] = \sum_{i=0}^{n} A_i (T(\mathrm{K}))^{-i} \qquad \text{(eq. S5)}$$

the coefficients A_i obtained are listed in table S5 together with the average relative deviations obtained for each IL solvent. These values give an overview of the precision of the experimental data which is in the present case always better than 1.2 %. The experimental solubility (expressed as molar fraction) is represented in Figure S12 as a function of the experimental temperatures studied.

, expressed on K_H, along with the percent average relative deviation of the fit (ARD)

**IL**	**A_0**	**A_1**	**ARD / %**
$[(Np)C_1im][NTf_2]$	8.52 ± 0.01	- 1263 ± 4	± 0.09
$[(SiC)C_1im][NTf_2]$	8.33 ± 0.06	- 1238 ± 18	± 0.6
$[(SiCSiC)C_1im][NTf_2]$	8.26 ± 0.09	- 1317 ± 27	± 0.6
$[(SiCSiC)C_1im][N(CN)_2]$	8.52 ± 0.12	- 1218 ± 39	± 0.9
$[(SiCSiC)C_1im][B(CN)_4]$	8.11 ± 0.04	- 1225 ± 13	± 0.2
$[(SiOSiC)C_1im][B(CN)_4]$	8.19 ± 0.17	- 1280 ± 54	± 1
$[(SiOSiC)C_1im][N(CN)_2]$	8.56 ± 0.02	- 1251 ± 5	± 0.09
$[(SiOSiC)C_1im][NTf_2]$	7.60 ± 0.04	- 1117 ± 11	± 0.4

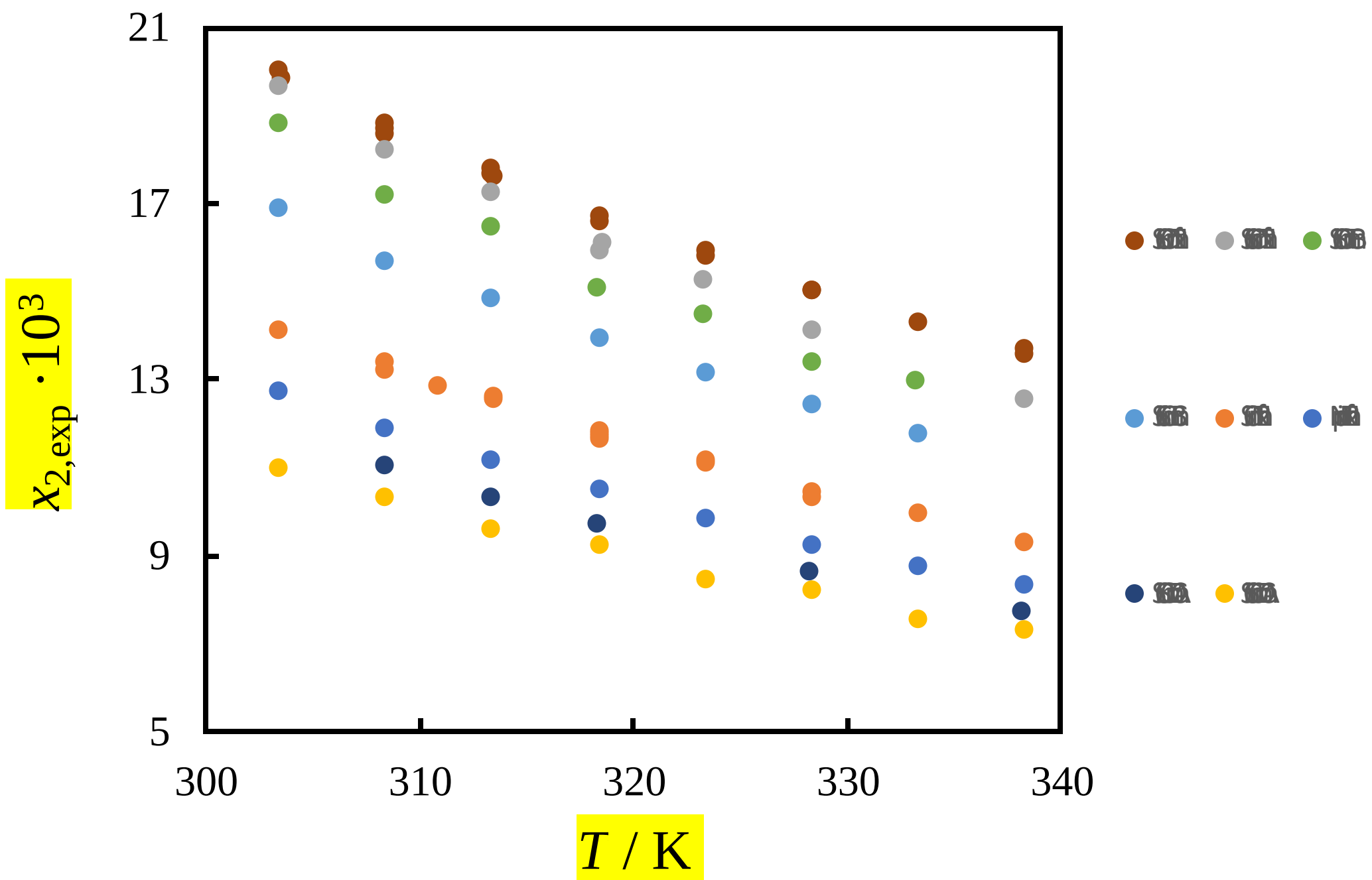

Figure S12. Mole fraction gas solubility of ethylene in the studied ionic liquids.

4.4. Thermodynamic properties derived from gas solubility measurements

The Gibbs energy of solvation, $\Delta_{sol}G^{\infty}$, here treated as the change in Gibbs energy when ethylene is transferred from the pure gas state at the standard pressure (p^{o} = 0.1 MPa) to its infinitely diluted state in the ionic liquid, can be calculated as follows:

$$\Delta_{sol}G^{\infty} = R \cdot T \cdot \ln\left(\frac{K_H}{p^o}\right) \qquad \text{(eq. S6)}$$

The enthalpy and entropy of solvation at infinite dilution were derived from the variation of the solubility with temperature (expressed in Henry's law constant) as described in equations S7 and S8. The results regarding the three thermodynamic properties of solvation are presented in table S6 for the different temperatures and the different ionic liquids studied in this work. In figures S13 and S14 the enthalpy and entropy of solvation of ethylene at infinite dilution are presented as a function of the temperature.

$$\Delta_{sol}H^{\infty} = -T^2 \frac{\partial}{\partial T}\left(\frac{\Delta_{sol}G^{\infty}}{T}\right) = -\mathrm{R} \cdot T^2 \cdot \frac{\partial}{\partial T}\left[ln\left(\frac{K_H}{p^o}\right)\right], \qquad \text{(eq. S7)}$$

$$\Delta_{sol}S^{\infty} = \frac{\Delta_{sol}H^{\infty} - \Delta_{sol}G^{\infty}}{T} = -\mathrm{R} \cdot T \cdot \frac{\partial}{\partial T}\left[ln\left(\frac{K_H}{p^o}\right)\right] - \mathrm{R} \cdot ln\left(\frac{K_H}{p^o}\right). \qquad \text{(eq. S8)}$$

Table S6. Thermodynamic functions of solvation of ethylene in the studied ionic liquids

T / K	$\Delta_{sol}G^{\infty\,a}$ / kJ·mol^{-1}	$\Delta_{sol}H^{\infty\,b}$ / kJ·mol^{-1}	$\Delta_{sol}S^{\infty\,c}$ / J·mol^{-1}·K^{-1}
	**[(Np)C$_1$im][NTf$_2$]**		
303.45	10.997	-9.4	-67.3
308.43	11.346	-9.7	-68.3
313.40	11.698	-10.0	-69.4
318.39	12.051	-10.4	-70.4
323.35	12.404	-10.7	-71.4
328.36	12.767	-11.0	-72.4
333.30	13.108	-11.4	-73.4
338.27	13.459	-11.7	-74.4
Uncertainty	± 0.007	± 0.3	± 0.9
	**[(SiC)C$_1$im][NTf$_2$]**		
303.45	10.73	-9.3	-65.8
308.43	11.07	-9.6	-66.8
308.43	11.05	-9.6	-66.8
310.91	11.24	-9.7	-67.3
313.41	11.38	-9.9	-67.7
313.42	11.39	-9.9	-67.8
318.38	11.76	-10.2	-68.9
318.39	11.73	-10.2	-68.8
318.39	11.75	-10.2	-68.8

T / K	$\Delta_{sol}G^{\infty\,a}$ / kJ·mol^{-1}	$\Delta_{sol}H^{\infty\,b}$ / kJ·mol^{-1}	$\Delta_{sol}S^{\infty\,c}$ / J·mol^{-1}·K^{-1}
318.39	11.77	-10.2	-68.9
323.36	12.08	-10.5	-69.8
323.37	12.08	-10.5	-69.8
328.35	12.46	-10.9	-70.9
328.36	12.44	-10.9	-70.8
333.34	12.77	-11.2	-71.7
338.30	13.15	-11.5	-72.8
Uncertainty	± 0.03	± 0.3	± 0.9
**[(SiCSiC)C$_1$im][NTf$_2$]**			
303.44	9.90	-9.8	-65
308.43	10.26	-10.2	-66
313.38	10.57	-10.5	-67
318.39	10.94	-10.8	-68
318.56	10.92	-10.8	-68
323.32	11.23	-11.2	-69
328.36	11.62	-11.5	-70
338.33	12.30	-12.2	-72
Uncertainty	± 0.04	± 0.4	± 1
**[(SiCSiC)C$_1$im][N(CN)$_2$]**			
303.45	11.37	-9.1	-67
308.42	11.71	-9.4	-68
313.40	12.09	-9.7	-69
318.38	12.39	-10.0	-70
323.36	12.81	-10.3	-71
328.34	13.10	-10.6	-72
333.31	13.53	-10.9	-73
338.31	13.81	-11.3	-74
Uncertainty	± 0.07	± 0.7	± 2
**[(SiCSiC)C$_1$im][B(CN)$_4$]**			
303.45	10.28	-9.3	-64
308.42	10.64	-9.6	-66
313.40	10.95	-9.9	-66
318.38	11.30	-10.2	-68
323.35	11.63	-10.5	-69
328.33	11.97	-10.9	-69
333.28	12.30	-11.2	-70
Uncertainty	± 0.02	± 0.4	± 1
**[(SiOSiC)C$_1$im][B(CN)$_4$]**			
303.45	10.00	-10	-65
308.42	10.41	-10	-66
313.39	10.68	-10	-67
318.37	11.09	-11	-68
323.33	11.37	-11	-69
328.32	11.76	-11	-70

***T* / K**	**$\Delta_{sol}G^{\infty}$ [a] / kJ·mol^{-1}**	**$\Delta_{sol}H^{\infty}$ [b] / kJ·mol^{-1}**	**$\Delta_{sol}S^{\infty}$ [c] / J·mol^{-1}·K^{-1}**
333.27	12.03	-12	-71
Uncertainty	± 0.07	± 1	± 3
	**[(SiOSiC)C_1im][N(CN)$_2$]**		
308.42	11.540	-9.5	-68
313.39	11.895	-9.8	-69
318.36	12.252	-10.1	-70
328.31	12.960	-10.7	-72
338.23	13.659	-11.4	-74
Uncertainty	± 0.007	± 0.4	± 1
	**[(SiOSiC)C_1im][NTf_2]**		
303.45	9.86	-8.5	-60.3
303.49	9.87	-8.5	-60.4
308.41	10.17	-8.7	-61.3
308.43	10.19	-8.7	-61.4
308.43	10.21	-8.7	-61.4
313.40	10.49	-9.0	-62.2
313.41	10.50	-9.0	-62.3
313.41	10.51	-9.0	-62.3
318.38	10.82	-9.3	-63.2
318.40	10.83	-9.3	-63.3
323.35	11.11	-9.6	-64.1
323.36	11.14	-9.6	-64.1
328.33	11.45	-9.9	-65.0
328.36	11.45	-9.9	-65.0
333.31	11.75	-10.2	-65.9
338.26	12.08	-10.5	-66.8
338.32	12.05	-10.5	-66.7
Uncertainty	± 0.03	± 0.3	± 0.9

[a] The uncertainty of the Gibbs free energy of solvation is an estimation of the confidence interval at a confidence level of 95% (± 2·σ), reflecting the dispersion of the experimental results; [b] The uncertainty of the enthalpy of solvation is an estimation of the confidence interval at a confidence level of 95%, reflecting the propagation of the uncertainty associated to the derivative of the Gibbs energy of solvation and precision of temperature measurements; [c] The uncertainty of the entropy of solvation is an estimation of the confidence interval at a confidence level of 95%, reflecting the propagation of the uncertainty associated to the Gibbs energy of solvation, the enthalpy of solvation and precision of temperature measurements.

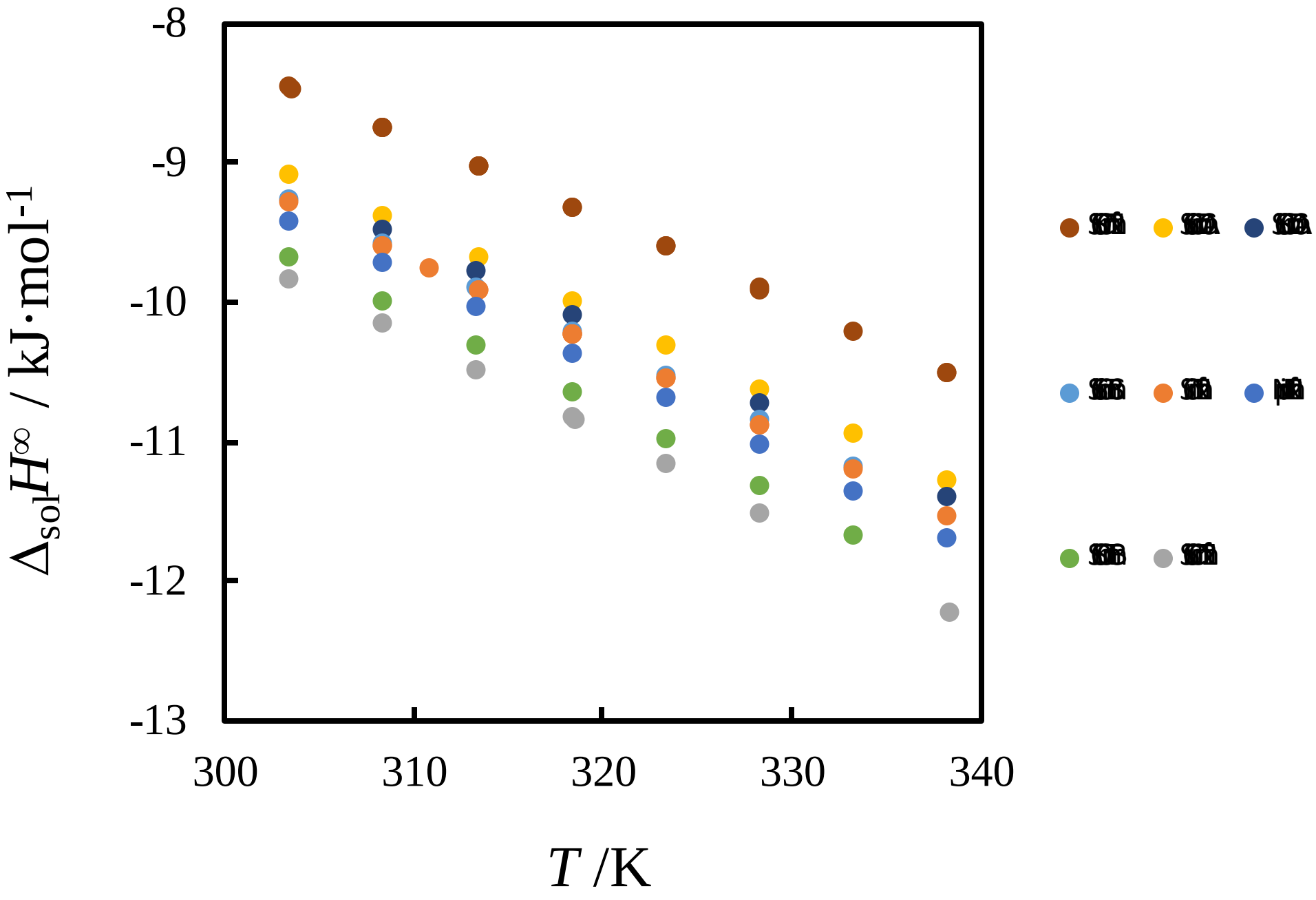

Figure S13. Molar enthalpy of solvation of ethylene at infinite dilution in the studied ionic liquids.

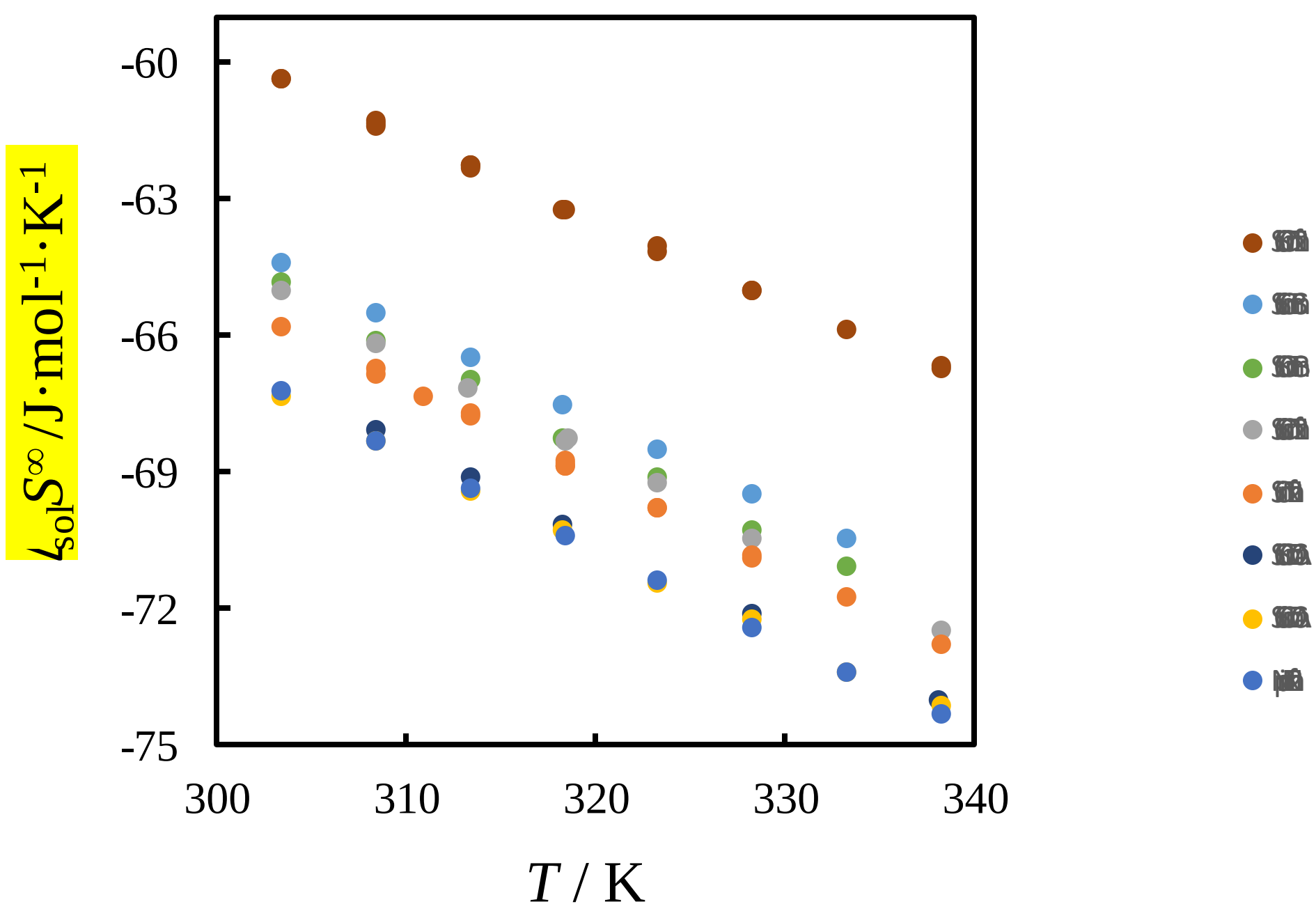

Figure S14. Molar entropy of solvation of ethylene at infinite dilution in the studied ionic liquids.

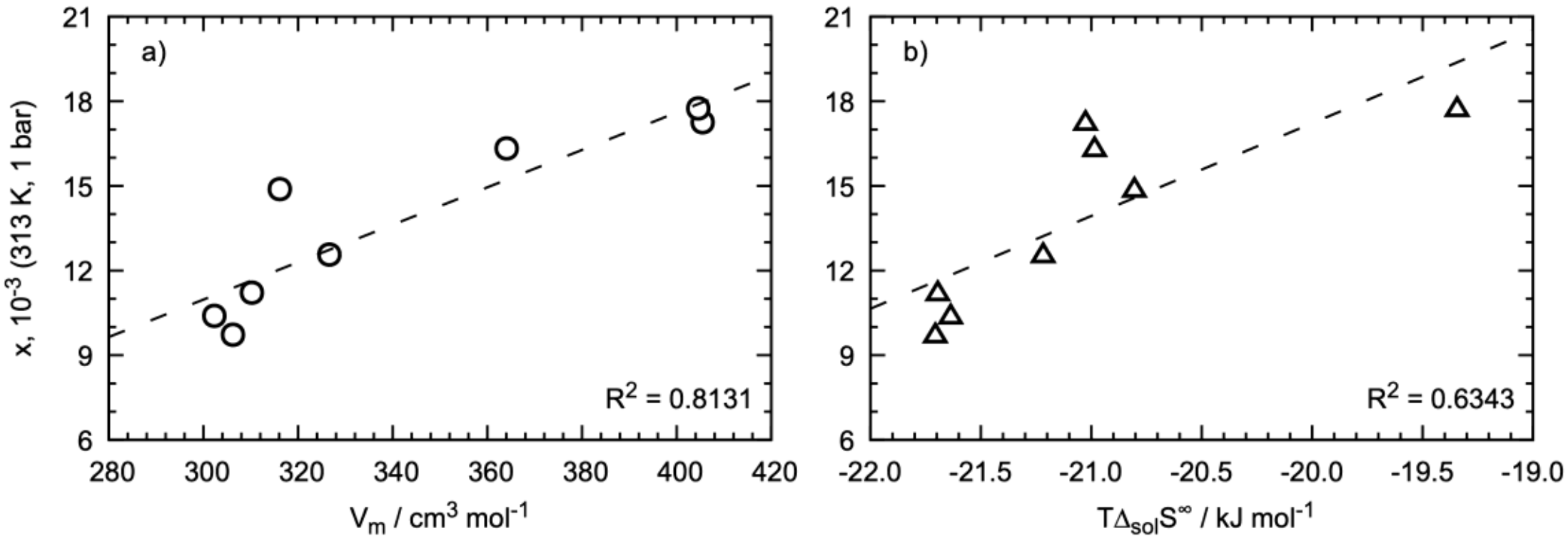

Figure S15. Correlation between the experimental mole-fraction solubility of ethylene in the studied ionic liquids at 313 K and 0.1 MPa and: (a) the molar volume of the ionic liquid,[1] used here as an experimentally accessible descriptor related to free volume and cavity-forming ability;[1] and (b) the entropic contribution to ethylene solvation derived from the temperature dependence of the Henry's law constants. The dashed lines are linear regressions intended only as guides to the eye.

References

1 [illegible]

2 [illegible]

3 [illegible]

4 [illegible]

5 [illegible]

6 [illegible]

7 [illegible]

8 [illegible]

9 [illegible]

10 [illegible]

11 [illegible]

12 [illegible]

13 [illegible]

14 [illegible]

15 [illegible]

16 [illegible]

17 [illegible]

18 [illegible]

19 [illegible]

20 [illegible]

21 [illegible]

22 [illegible]

23 [illegible]

24 [illegible]

25 [illegible]

26 [illegible]

27 [illegible]

28 [illegible]

29 [illegible]

30 J[illegible]

31 L[illegible]

32 R[illegible]

33 Y[illegible]
